# Supplementary material for: Age-progressive interplay of HSP-proteostasis, ECM-cell junctions and biomechanics ensures C. elegans astroglial architecture
Source: Nat Commun. 2024 Apr 3;15:2861. doi: 10.1038/s41467-024-46827-2 (PMC10991496; doi:10.1038/s41467-024-46827-2)
Supplement: Supplementary file 1 — Supplementary Information [file 41467_2024_46827_MOESM1_ESM.pdf]

Supplementary Figures & Legends

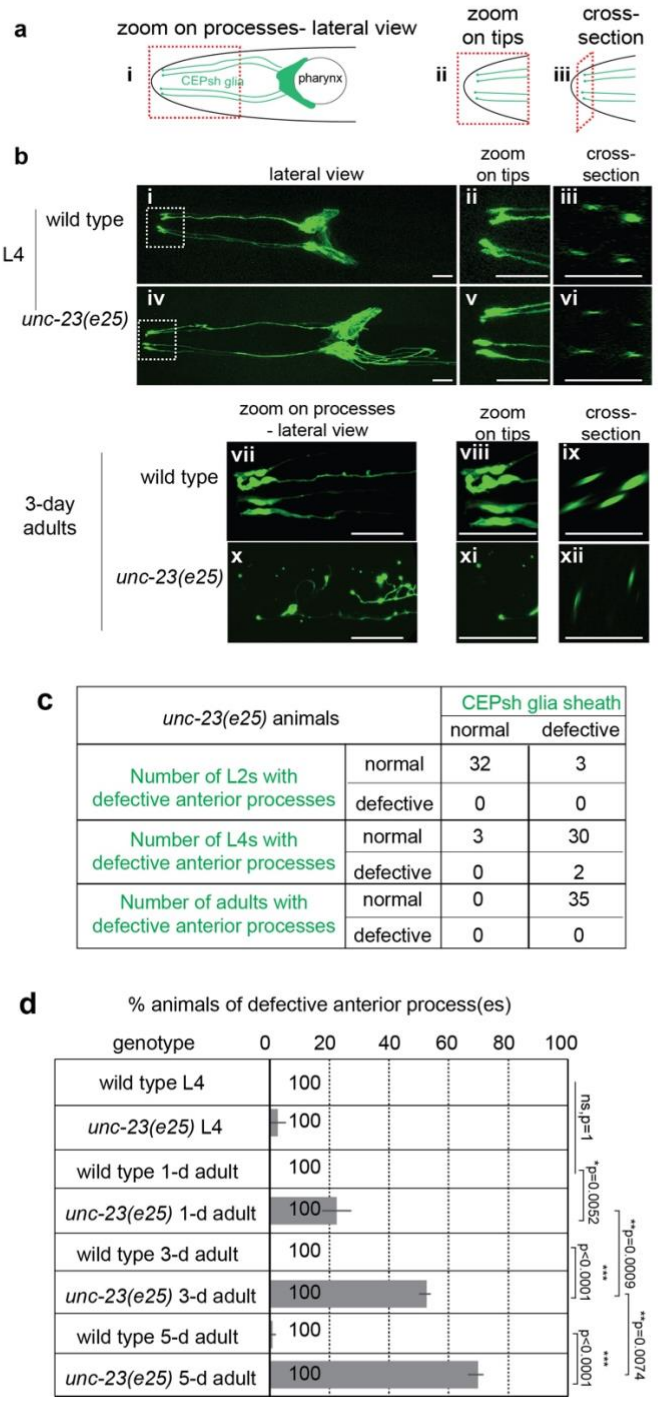

Supplementary Fig. 1. Anterior processes of CEPsh glia are defective in *unc-23* mutant adults but not in L4 stage. (a-b) Each of the 4 CEPsh glia (green) has an anterior process and tip arriving to the sensory endings of the *C. elegans* nose. These are shown here in lateral view of entire CEPsh glia (ai, bi,

biv,) or lateral view of their anterior glia processes (aii, bvii, bx) or zoom view of their tips (aiii, bviii, bxi) or in cross-sectional view of their tips (aiii, biii, bvi, bix, bxii). Anterior processes and tips of CEPsh glia are largely not defective in *unc-23* mutants compared to wild-type animals in L4 stage (bi-vi), but they present defects of mispositioning and fragmentation in adult stages (Bvii-Bxi). (c-d) These CEPsh glia defects appear in significant number of adults *unc-23* mutant animals (c) and they are age-progressive (d). Scale bars, 10μm. Animal axes as in Figure 1. n= 3 independent experiments with total animal numbers noted inside bar graphs (d). Data are presented as mean values +/- SD, ***p-value<0,0001, **p-value<0,001, *p-value<0,01, unpaired t-test. Source data and p values are presented in the provided Source Data file.

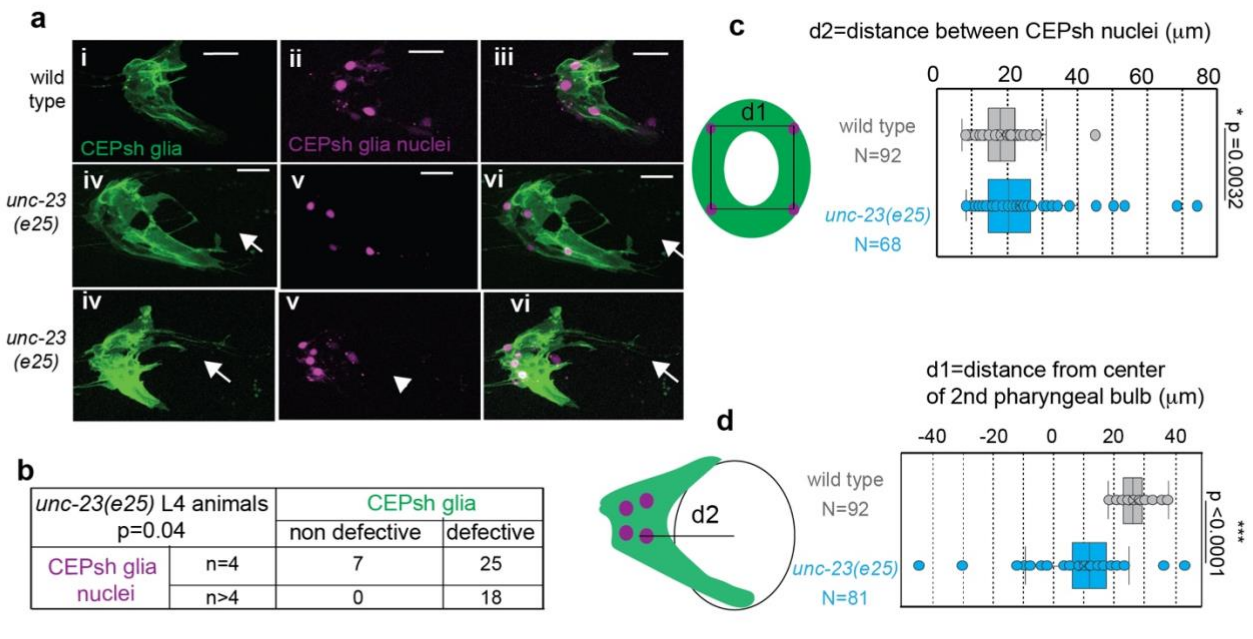

Supplementary Fig. 2. *Unc-23* mutant L4 animals present fragmentation and mispositioning of CEPsh glia nuclei. (a-c). Compared to wild-type animals L4 *unc-23* mutant animals with established defects in CEPsh glia membrane sheath (green) may present fragmentation of CEPsh glia nuclei (magenta) (a-b). CEPsh glia also present abnormal placement of nuclei between each other (c) and relative to the second pharyngeal bulb (d). This is in contrast to L2 *unc-23* mutant animals that present no defects in CEPsh glia nuclei (Figure 2). Total animal numbers noted as N for each genotype. *, p-value<0,01, unpaired t-test. Data are presented as mean values +/- SD. Dots, limits, lines, whiskers in box-whisker plots as defined in Fig. 1. Source data and p values are presented in the provided Source Data file.

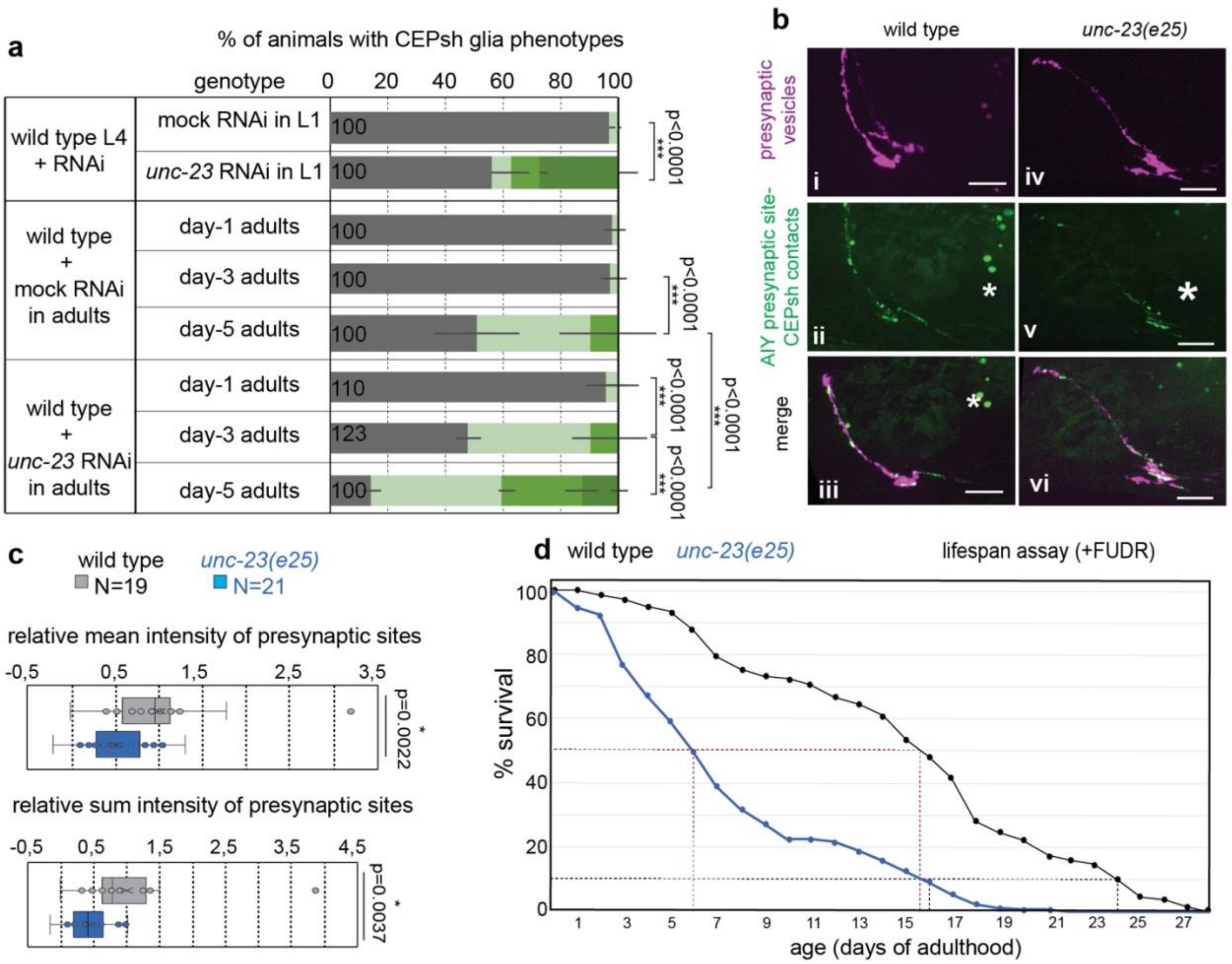

Supplementary Fig. 3. UNC-23/BAG2 acts in larval and adult stages and affects synapse aging and animal lifespan. (a). Subjecting wild-type adults in *unc- 23* RNAi results in significant defects in CEPsh glial cell architecture, that worsen progressively throughout days 1 and 5 of adulthood. Total animal numbers are noted inside histogram bars. two-way Fisher's exact test performed. (b-c). In five-day old adults, AIY presynaptic vesicles (by RAB-3::mCherry marker, magenta) have decreased mean and sum intensity in *unc-23* mutants (ai-iii,b) compared to wild-type animals (biv-vi, c). *, gut autofluorescence. n=12 animals per genotype. unpaired t-test. (a, c) Error bars represent mean ± standard deviation. Number of independent experiments for each condition is n=3, unless otherwise noted. Dots, limits, lines, whiskers in box-whisker plots as defined in Fig. 1. Total number of animals per condition, number of independent experiments, primary data, and statistical analysis (including 2-

way ANOVA) are presented in the Source Data. (d). *unc-23* mutants show decreased lifespan compared to wild-type animals (both strains express CEPsh glia::myrGFP). The median lifespan (50% survival) is 15,5 days for wild-type worms and 6 days for *unc-23* mutants (both with integrated array expressing CEPsh glia::myrGFP) and the maximum lifespan (5% survival) is 24 days and 16 days of these two populations respectively. Lifespan assays were performed with the use of 50uM FuDR. n= 3 independent experiments with total animal numbers noted for each condition inside bar graphs (a) or noted as N in box-whisker plots (c) or in Supplementary Table 1 (d). Result analysis and statistics were performed using the Kaplan–Meier method and Wilcoxon rank sum test. p value<0,001. Primary source data (presented as mean values +/- SD) are provided for 3a-c in the Source Data file and for 3d in Supplementary Table 1.

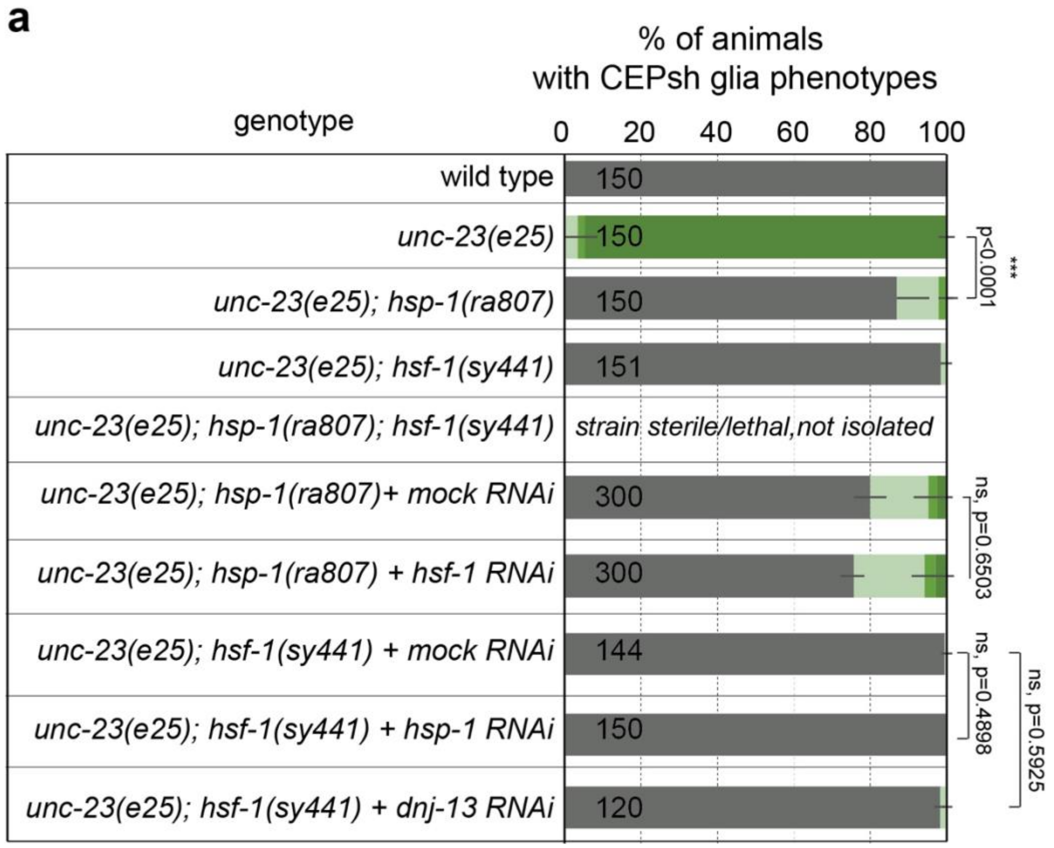

Supplementary Fig. 4. HSP-1 and DNJ-13 can affect roles of UNC-23/BAG2 in CEPsh glial integrity independently of HSF-1 activation. CEPsh glia defects (as presented in schematics in Fig. 4a) in *unc-23* mutants are suppressed when also mutating *hsf-1*, suggesting that HSF-1 affects UNC-23/BAG2 roles in CEPsh glial integrity. Post-embryonic RNAi knock down of HSP-1 or DNJ-13 suppresses the CEPsh glia defects, even in absence of HSF-1, suggesting they can act independently of HSF-1 activation in this context. n= 3 independent experiments with total animal numbers noted inside the bar graph. Number or animals noted in histogram, number of independent experiments is n=3. Total number of animals per condition, number of independent experiments, primary data, and statistical analysis (including 2-way ANOVA) are presented in the Source Data.

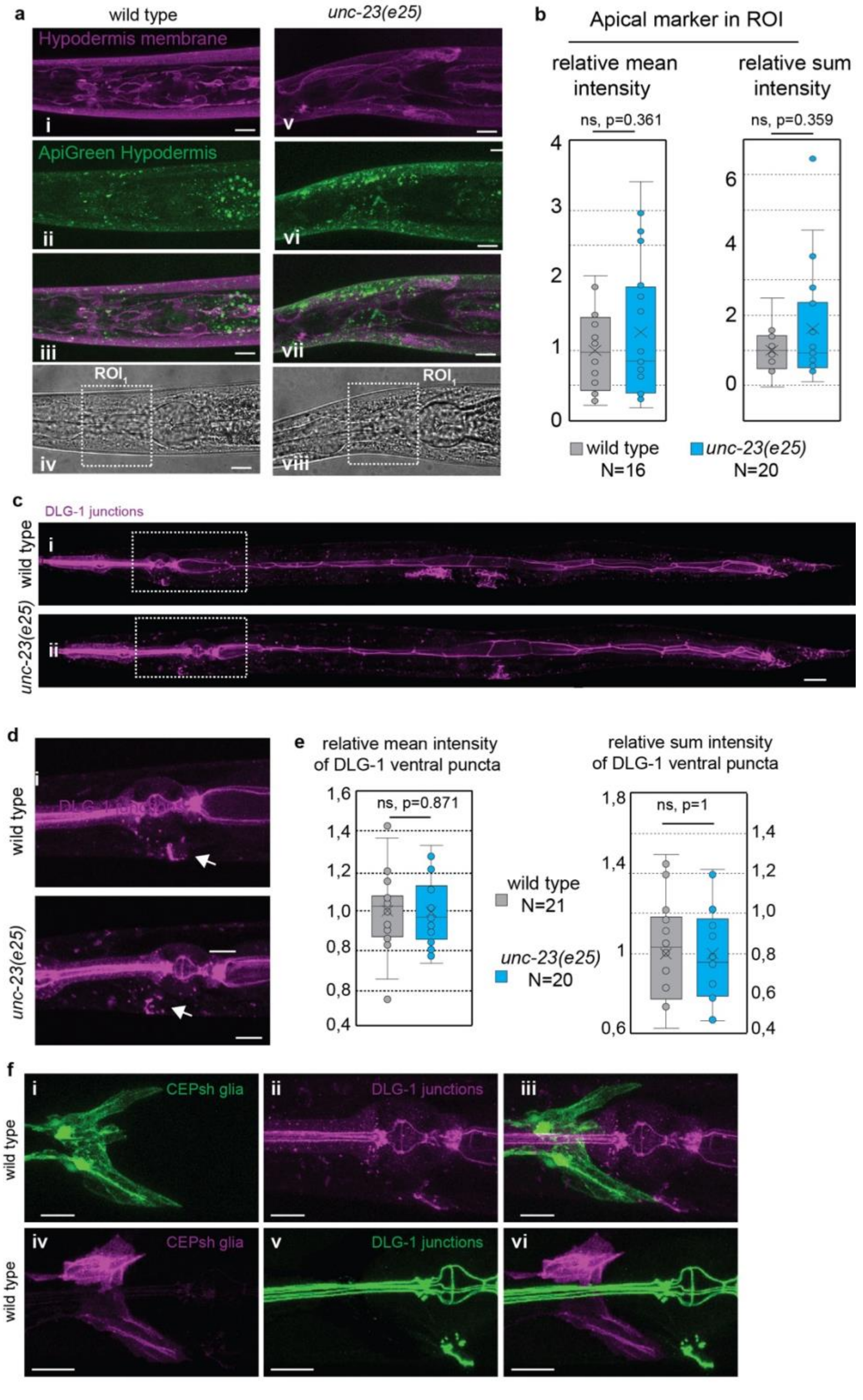

Supplementary Fig. 5. UNC-23/BAG2 acts for glial polarity independently of epithelial polarity and integrity. Overall epithelial integrity is not disrupted in *unc-23* mutants, as assessed by epithelial

membranes labeling across the body (magenta) (ai,av,b), and quantitative analysis of the epithelial apical domain marker ApiGreen (green), neighboring the CEPsh glia (aii,avi,b). **C-E.** Epithelial integrity of DLG-1 junctions (magenta) throughout the body is not disrupted in *unc-23* mutants (c) and areas of DLG-1 enrichments are comparable in *unc-23* mutants and wild-type animals (d-e). **f)** Epithelial DLG-1 junctions maintain close proximity to the posterior edge of the CEPsh glia membrane sheath in wild-type animals, as assessed by epithelial DLG-1 (fi-iii) or endogenously-tagged DLG-1. The doted squares in A are regions of interest (ROI) in b, quantified as described in Methods. The doted squares in c are d panels. Molecular reporters used as listed in Methods and Supplementary Tables 4,5. Total animal numbers noted as N in each genotypes in box-whisker plots (b, e). *, p-value<0,01, unpaired t-test. ns, non-significant. Arrows, DLG-1 ventral enrichment. Scale bars, 10μm. Dots, limits, lines, whiskers in box-whisker plots and animal axis in images as defined in Fig. 1. Data are presented as mean values +/- SD. Source data and p values are presented in the provided Source Data file.

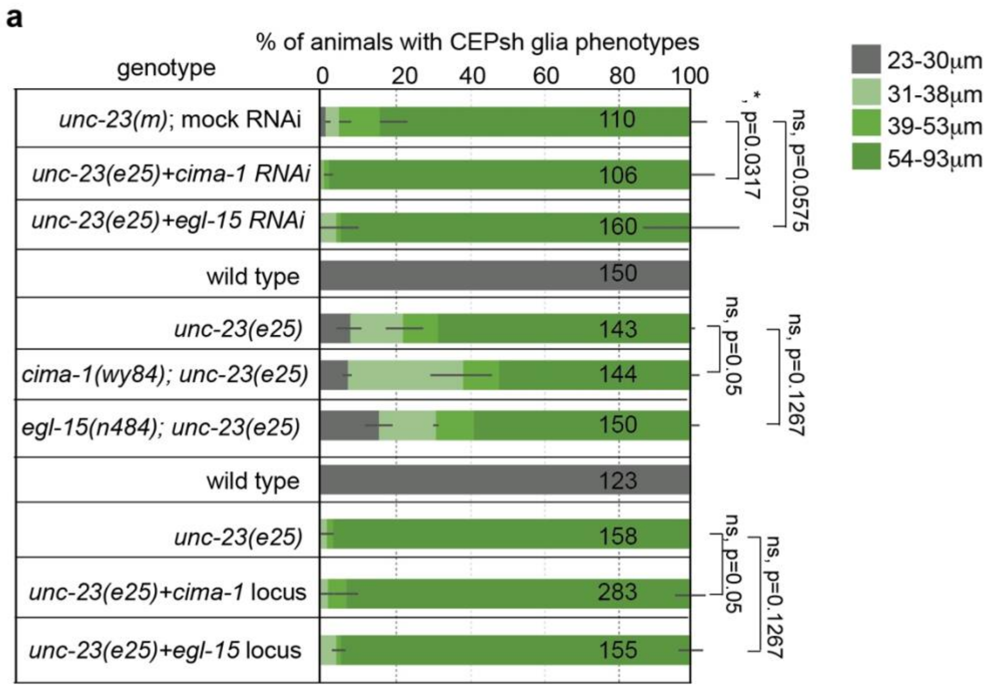

Supplementary Fig. 6. UNC-23/BAG2 acts for CEPsh glia integrity independently of epithelial allostery cues. (a) RNAi-mediated knock-down (KD) of epithelial *allostery cues* EGL-15/FGFR and CIMA-1/ SLC17A5 (as defined by [1]), or their genetic mutants or their overexpression by fosmid expression (locus) do not suppress the CEPsh glia defects in *unc-23* mutants (RNAi of *cima-1* appears to enhance the CEPsh glia defects in *unc-23* mutants). * p value< 0,001. Error bars, mean ± standard deviation. n= 3 independent experiments, total animal numbers noted in the bar graph. Two-way Fisher's exact test performed. Total number of animals per condition, number of independent experiments, primary data, and statistical analysis (including 2-way ANOVA) are presented in the Source Data.

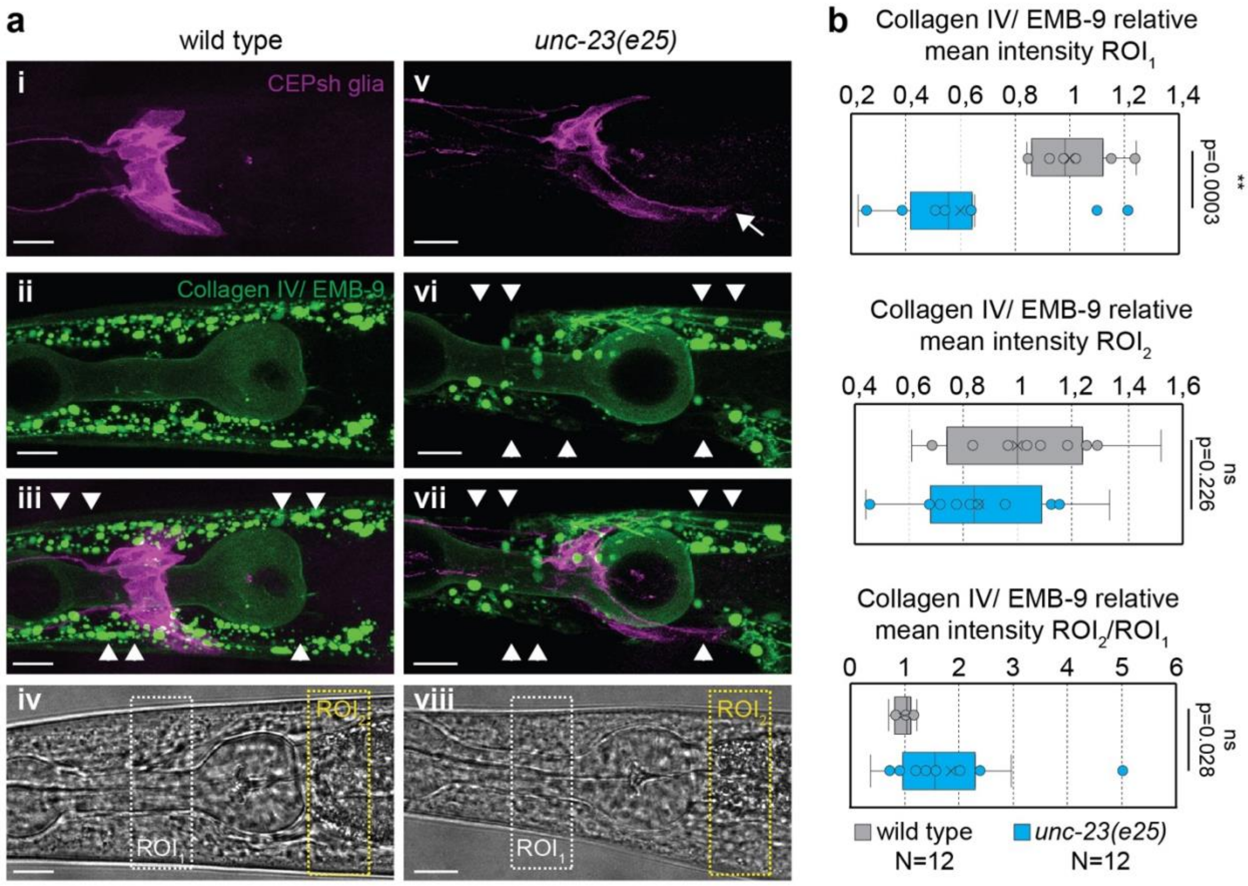

Supplementary Fig. 7. Collagen IV/ EMB-9 localization is impaired in *unc-23* mutants.

(a-b). EMB-9 (green) localization is impaired in *unc-23* mutants compared to wild-type animals, accumulating in the posterior part of CEPsh glia (magenta). Scale bars, 10μm. Arrows, CEPsh glia defects. Arrowhead, EMB-9 defects. ROI_1, ROI_2 correspond in A-D. Animal axes as in Figure 1. Total animal numbers noted as N with genotypes in box-whisker plots. **, p-value<0,001, *, p-value<0,05, unpaired t-test. ns, non-significant. Data are presented as mean values +/- SD. Dots, limits, lines, whiskers in box-whisker plots as defined in Fig. 1. Number of animals, number of independent experiments, primary data, and statistical analysis (including 2-way ANOVA) are presented in the Source Data.

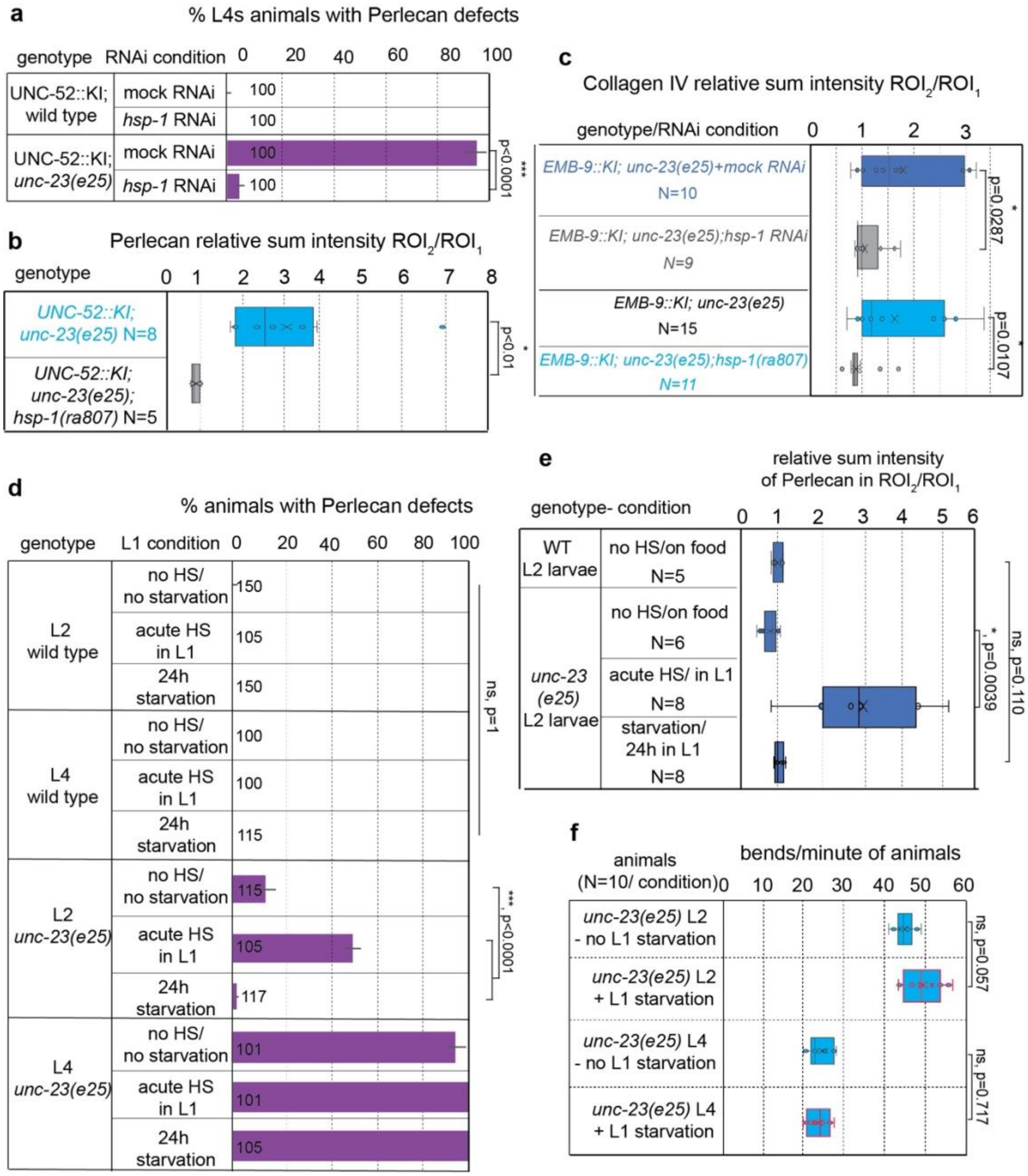

Supplementary Fig. 8. ECM defects in *unc-23* mutants are affected by HSP-1, heat and caloric restriction but do not result from locomotion differences. (a-c) *hsp-1* knock-down by RNAi or *hsp-1(ra807)* mutation suppresses the localization defects of Perlecan/UNC-52 (a-b) and Collagen/ EMB-9 in *unc-23* mutant L4 animals (c). This is quantified by the % of animals in populations of different

genotypes/conditions that present the Perlecan/UNC-52 defects in *unc-23* mutants (in Supplementary Fig. 8a), quantified as per Fig. 5a, or by quantifications of relative sum intensity of Perlecan/UNC-52 content in ROI_2/ROI_1 (posterior/anterior of glia) (in Supplementary Fig. 8b) as initially quantified in Fig. 5b-c. The Collagen/ EMB-9 localization is examined using quantification of its relative sum intensity in the ROI_2/ROI_1 (posterior/anterior of glia) (in Supplementary Fig. 8b), as initially quantified in Supplementary Fig. 7d. (d-e) The low defects of Perlecan/UNC-52 observed in L2 animals of *unc-23* mutants are enhanced by subjecting animals to acute temperature-increase and suppressed by subjecting animals to starvation (at the L1 stage). Defects of Perlecan/UNC-52 in L4 animals of *unc-23* mutants are similar in all conditions. No such Perlecan/UNC-52 defects are observed in wild-type animals subject to these conditions of temperature increase of starvation. (f) L2 or L4 animals of *unc-23* mutants present similar locomotion (as quantified by body bends per minute) in normal conditions or after starvation at L1 stage. n= 3 independent experiments with total animal numbers noted inside bar graphs for bar graphs (a, d) or noted as N with genotypes in box-whisker plots (b, c, e, f). Two-sided unpaired t-test, p values as in Fig. 2. t-test p-values: ***, <0,001; **, <0,005; *, <0,05, ns, non-significant. Error bars represent mean ± standard deviation. Total animal numbers are noted inside histogram bars or dot-plot graphs. Number of independent experiments for each condition is n=3, unless otherwise noted. Dots, limits, lines, whiskers in box-whisker plots are defined as in Fig. 1. Number of animals and number of independent experiments, primary data, and statistical analysis (including 2-way ANOVA analysis) are presented in the Source Data. Dots, limits, lines, whiskers, error bars in box-whisker and bar graphs are defined as in Fig. 2.

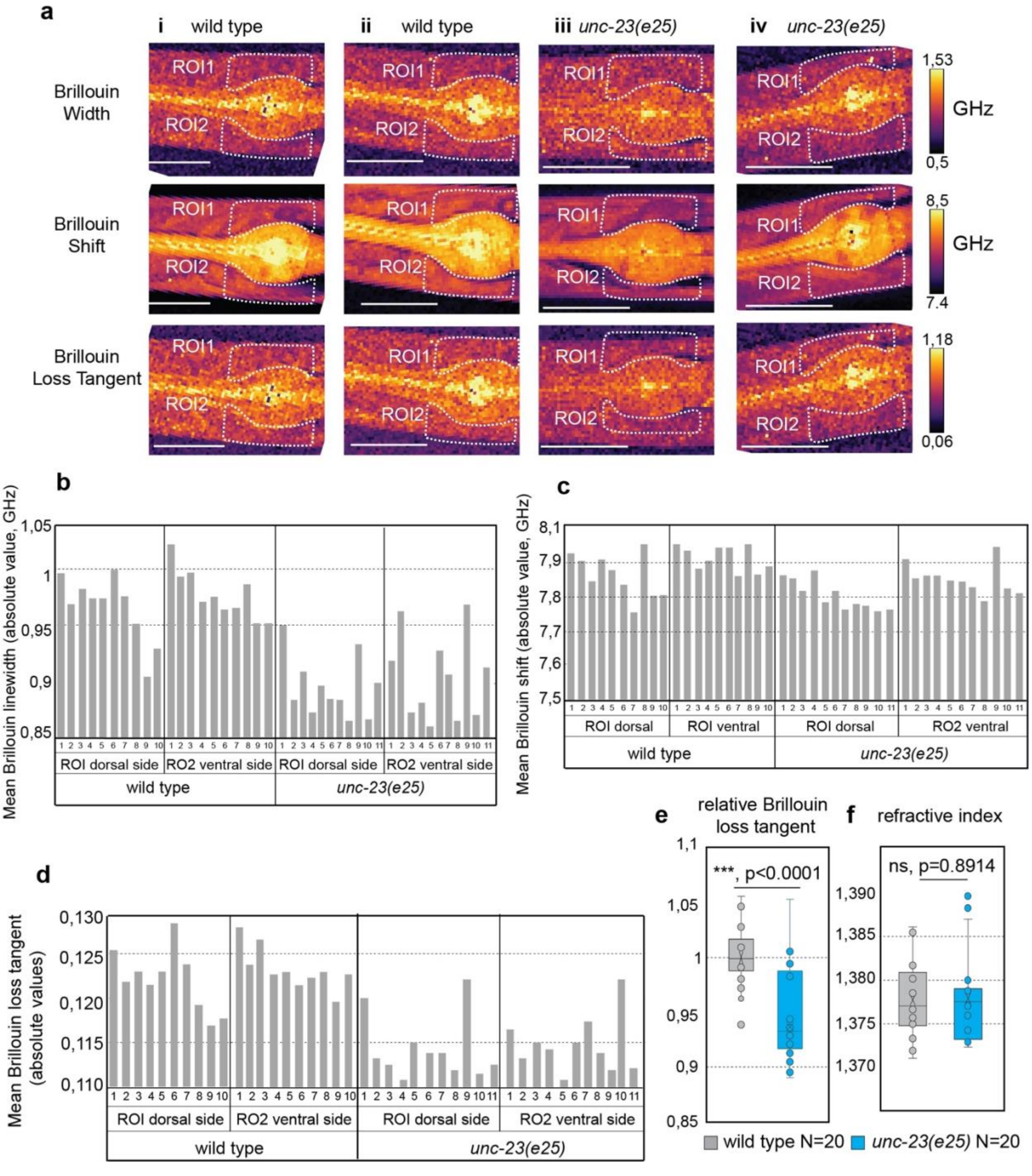

Supplementary Fig. 9. Brillouin width, shift, loss tangent and refractive index measurements of *unc-23* mutants and wild-type animals. (a) Representative Brillouin microscopy images showing the Brillouin width/ viscous contrast ("viscosity"), Brillouin shift/ elastic contrast ("elasticity") and Brillouin loss tangent (deconvolved for the instrument response, see Methods) in L2 individuals of wild-type animals and *unc-23*

mutants. Depicted ROIs are in regions neighboring CEPsh glia localization (see Methods). Scale bars, 10μm. White dotted rectangles in (A), ROIs in (B, C) as in Fig. 7. (b-c) Quantification of the tissue ROIs show decreased Brillouin width/ viscous contrast ("viscosity") and decreased Brillouin shift/ elastic contrast ("elasticity") of *unc-23* mutants compared to wild-type animals. Absolute values (average±SD) of mean Brillouin width (for viscosity) are 0,9712±0,0274 and 0,9122±0,0443 after deconvolution, in wild-type and *unc-23* mutant animals, respectively. Relative values (average±SD) of mean Brillouin width are 1,0000±0,0283 and 0,9393±0,0456 for wild-type and mutant animals, respectively, presented in Fig. 7f. The values of *unc-23* mutant and wild-type animals are significantly different, p value < 0.0001. Absolute values (average±SD) of mean Brillouin shift are 7,9106±0,0548 and 7,8380±0,0756 for wild-type and *unc-23* mutant animals, respectively. Relative values (average±SD) of mean Brillouin shift are 1,0000±0,0069 and 0,9908±0,0096 for wild-type and mutant animals, respectively, presented in Fig. 7f. These values are significantly different with p value<0.005. (d-e) Absolute and relative values of Brillouin loss tangent represent viscoelasticity, independently of the sample's refractive index. The Brillouin loss tangent is significantly lower in *unc-23* mutants compared to wild-type animals (p value < 0.0001). Absolute values of mean loss tangent (Average±SD) are 0,1227±0,0033 and 0,1163±0,0056 for wild-type and mutant animals, respectively. Relative values of mean loss tangent (Average±SD) are 1,0000±0,0268 and 0,9476±0,0455 for wild-type and mutant animals, respectively. (f) Absolute values of refractive index in tissue ROIs of *unc-23* mutants and wild-type animals as assessed by commercial holotomography are not significantly different (p value= 0.8386). b-f) n ≥20 ROIs (≥10 animals). unpaired t-test. Data are presented as mean values +/- SD. Total animal numbers noted as N with genotypes, inside box-whisker plots (e, f). Source data and exact p values are presented in the provided Source Data file.

Supplementary Table 1. Primary data of lifespan assays.

This table presents the primary data of the lifespan assays. Lifespan assays were performed with final >100 non-censored animals per genotype, in 3 parallel trials. Strains used were the wild-type and unc-23(e25) both expressing nsIs374(CEPsh::myrGFP) integrated array. The average of all 3 trials (see end of table, page 3) is presented in Supplementary Figure 3. Here all animal numbers (alive, dead, censored) are presented as well as % average, stdev.

	WT=nsIs374= P[CEPsh}::myrGFP						*unc-23(e25); nsIs374= P[CEPsh}::myrGFP*					
					trial 1						trial 1	
D	Alive adults	Dead adults	Censored adults	all dead until day	% dead until this day	% alive until this day	Alive adults	Dead adults	Censored adults	all dead until day	% dead until this day	% alive this day
0	120	0	76	0	0,0%	100,0%	120	0	18	0	0,0%	100,0%
1	44	0	4	0	0,0%	100,0%	95	7	2	7	8,5%	91,5%
2	39	1	6	1	4,2%	95,8%	92	1	7	8	9,8%	90,2%
3	33	0	0	1	4,2%	95,8%	72	13	0	21	25,6%	74,4%
4	32	1	1	2	8,3%	91,7%	67	5	1	26	31,7%	68,3%
5	31	0	0	2	8,3%	91,7%	60	6	4	32	39,0%	61,0%
6	29	2	0	4	16,7%	83,3%	47	9	2	41	50,0%	50,0%
7	27	2	1	6	25,0%	75,0%	32	13	0	54	65,9%	34,1%
8	25	1	1	7	29,2%	70,8%	30	2	0	56	68,3%	31,7%
9	24	0	1	7	29,2%	70,8%	26	4	3	60	73,2%	26,8%
10	23	0	0	7	29,2%	70,8%	19	4	0	64	78,0%	22,0%
11	22	1	2	8	33,3%	66,7%	19	0	0	64	78,0%	22,0%
12	20	0	0	8	33,3%	66,7%	19	0	0	64	78,0%	22,0%
13	19	1	1	9	37,5%	62,5%	14	5	1	69	84,1%	15,9%
14	17	1	0	10	41,7%	58,3%	11	2	0	71	86,6%	13,4%
15	16	1	2	11	45,8%	54,2%	9	2	0	73	89,0%	11,0%
16	13	1	0	12	50,0%	50,0%	7	2	0	75	91,5%	8,5%
17	11	2	1	14	58,3%	41,7%	3	4	0	79	96,3%	3,7%
18	7	3	0	17	70,8%	29,2%	1	2	0	81	98,8%	1,2%
19	5	2	0	19	79,2%	20,8%	0	1	0	82	100,0%	0,0%
20	5	0	0	19	79,2%	20,8%	0	0	0	82	100,0%	0,0%
21	4	1	0	20	83,3%	16,7%	0	0	0	82	100,0%	0,0%
22	3	1	0	21	87,5%	12,5%	0	0	0	82	100,0%	0,0%
23	2	1	0	22	91,7%	8,3%	0	0	0	82	100,0%	0,0%
24	1	1	0	23	95,8%	4,2%	0	0	0	82	100,0%	0,0%
25	0	1	0	24	100,0%	0,0%	0	0	0	82	100,0%	0,0%
26	0	0	0	24	100,0%	0,0%	0	0	0	82	100,0%	0,0%
27	0	0	0	24	100,0%	0,0%	0	0	0	82	100,0%	0,0%
28	0	0	0	24	100,0%	0,0%						
M		24					SUM		82			

	WT=nsIs374= P[CEPsh}::myrGFP						*unc-23(e25); nsIs374= P[CEPsh}::myrGFP*					
						trial 2					trial 2	
D	ve adults	Dead adults	Censored adults	all dead until day	% dead until this day	% alive this day	Alive adults	Dead adults	Censored adults	all dead until day	% dead until this day	% alive this day
0	120	0	57	0	0,0%	100,0%	120	0	11	0	0,0%	100,0%
1	63	0	0	0	0,0%	100,0%	109	0	2	0	0,0%	100,0%
2	63	0	7	0	0,0%	100,0%	102	5	9	5	6,3%	93,7%
3	56	0	2	0	0,0%	100,0%	82	11	2	16	20,3%	79,7%

4	54	0	0	0	0,0%	100,0%	67	13	1	29	36,7%	63,3%
5	53	1	1	1	2,5%	97,5%	63	3	8	32	40,5%	59,5%
6	49	3	1	4	10,0%	90,0%	45	10	3	42	53,2%	46,8%
7	43	5	0	9	22,5%	77,5%	34	8	2	50	63,3%	36,7%
8	41	2	0	11	27,5%	72,5%	26	6	0	56	70,9%	29,1%
9	40	1	3	12	30,0%	70,0%	23	3	3	59	74,7%	25,3%
10	36	1	2	13	32,5%	67,5%	15	5	0	64	81,0%	19,0%
11	34	0	3	13	32,5%	67,5%	15	0	0	64	81,0%	19,0%
12	29	2	1	15	37,5%	62,5%	14	1	0	65	82,3%	17,7%
13	27	1	1	16	40,0%	60,0%	14	0	0	65	82,3%	17,7%
14	24	2	0	18	45,0%	55,0%	11	3	0	68	86,1%	13,9%
15	20	4	0	22	55,0%	45,0%	7	4	0	72	91,1%	8,9%
16	20	0	1	22	55,0%	45,0%	3	4	0	76	96,2%	3,8%
17	15	4	0	26	65,0%	35,0%	1	2	0	78	98,7%	1,3%
18	8	7	1	33	82,5%	17,5%	0	1	0	79	100,0%	0,0%
19	7	0	0	33	82,5%	17,5%	0	0	0	79	100,0%	0,0%
20	6	1	0	34	85,0%	15,0%	0	0	0	79	100,0%	0,0%
21	5	1	0	35	87,5%	12,5%	0	0	0	79	100,0%	0,0%
22	5	0	0	35	87,5%	12,5%	0	0	0	79	100,0%	0,0%
23	4	1	0	36	90,0%	10,0%	0	0	0	79	100,0%	0,0%
24	2	2	0	38	95,0%	5,0%	0	0	0	79	100,0%	0,0%
25	1	1	0	39	97,5%	2,5%	0	0	0	79	100,0%	0,0%
26	1	0	0	39	97,5%	2,5%	0	0	0	79	100,0%	0,0%
27	1	0	0	39	97,5%	2,5%	0	0	0	79	100,0%	0,0%
28	0	1	0	40	100,0%	0,0%	0	0		79	100,0%	0,0%
M		40					SUM		79			
	WT=nsIs374= P[CEPsh}::myrGFP						*unc-23(e25); nsIs374= P[CEPsh}::myrGFP*					
	trial 3						trial 3					
D	ve adults	Dead adults	Censored adults	all dead until day	% dead until this day	% alive this day	Alive adults	Dead adults	Censored adults	all dead until day	% dead until this day	% alive this day
0	120	0	40	0	0,0%	100,0%	120	0	24	0	0,0%	100,0%
1	80	0	2	0	0,0%	100,0%	91	5	3	5	6,9%	93,1%
2	78	0	10	0	0,0%	100,0%	88	0	5	5	6,9%	93,1%
3	66	2	0	2	4,3%	95,7%	72	11	1	16	22,2%	77,8%
4	65	1	1	3	6,5%	93,5%	66	5	5	21	29,2%	70,8%
5	63	1	2	4	8,7%	91,3%	52	9	0	30	41,7%	58,3%
6	60	1	2	5	10,9%	89,1%	48	4	1	34	47,2%	52,8%
7	56	2	2	7	15,2%	84,8%	42	5	2	39	54,2%	45,8%
8	53	1	3	8	17,4%	82,6%	32	8	1	47	65,3%	34,7%
9	48	2	7	10	21,7%	78,3%	28	3	3	50	69,4%	30,6%
10	40	1	0	11	23,9%	76,1%	22	3	1	53	73,6%	26,4%
11	40	0	0	11	23,9%	76,1%	21	0	0	53	73,6%	26,4%
12	39	1	0	12	26,1%	73,9%	20	1	2	54	75,0%	25,0%
13	38	1	2	13	28,3%	71,7%	17	1	0	55	76,4%	23,6%
14	34	2	0	15	32,6%	67,4%	15	2	0	57	79,2%	20,8%
15	29	5	0	20	43,5%	56,5%	13	2	0	59	81,9%	18,1%

16	27	2	1	22	47,8%	52,2%		11	2	0	61	84,7%	15,3%
17	23	3	1	25	54,3%	45,7%		8	3	0	64	88,9%	11,1%
18	19	3	1	28	60,9%	39,1%		4	4	0	68	94,4%	5,6%
19	17	1	0	29	63,0%	37,0%		2	2	0	70	97,2%	2,8%
20	15	2	0	31	67,4%	32,6%		1	1	0	71	98,6%	1,4%
21	12	3	0	34	73,9%	26,1%		1	0	0	71	98,6%	1,4%
22	12	0	0	34	73,9%	26,1%		0	1	0	72	100,0%	0,0%
23	12	0	0	34	73,9%	26,1%		0	0	0	72	100,0%	0,0%
24	9	3	0	37	80,4%	19,6%		0	0	0	72	100,0%	0,0%
25	4	5	0	42	91,3%	8,7%		0	0	0	72	100,0%	0,0%
26	4	0	0	42	91,3%	8,7%		0	0	0	72	100,0%	0,0%
27	1	3	0	45	97,8%	2,2%		0	0	0	72	100,0%	0,0%
28	0	1		46	100,0%	0,0%		0	0		72	100,0%	0,0%
M		46							72				
	% mean of all above, WT vs unc-23(25) with nsIs374												
	average + stdev, n=3												
		WT P[CEPsh}::myrGFP		*unc-23(e25)* P[CEPsh}::myrGFP									
D		verage % urvival (n=3)	stdev % survival (n=3)	Average % survival (n=3)	stdev % survival (n=3)								
0		100,0%	0,0%	100,0%	0,0%								
1		100,0%	0,0%	94,8%	4,5%								
2		98,6%	2,4%	92,3%	1,8%								
3		97,2%	2,5%	77,3%	2,7%								
4		95,0%	4,4%	67,5%	3,8%								
5		93,5%	3,5%	59,6%	1,3%								
6		87,5%	3,6%	49,9%	3,0%								
7		79,1%	5,1%	38,9%	6,1%								
8		75,3%	6,4%	31,8%	2,8%								
9		73,0%	4,5%	27,6%	2,7%								
10		71,5%	4,3%	22,4%	3,7%								
11		70,1%	5,2%	22,4%	3,7%								
12		67,7%	5,8%	21,6%	3,7%								
13		64,7%	6,2%	19,1%	4,0%								
14		60,2%	6,4%	16,1%	4,1%								
15		51,9%	6,1%	12,6%	4,8%								
16		49,1%	3,7%	9,2%	5,8%								
17		40,8%	5,4%	5,3%	5,1%								
18		28,6%	10,8%	2,3%	2,9%								
19		25,1%	10,4%	0,9%	1,6%								
20		22,8%	9,0%	0,5%	0,8%								
21		18,4%	7,0%	0,5%	0,8%								
22		17,0%	7,8%	0,0%	0,0%								
23		14,8%	9,8%	0,0%	0,0%								
24		9,6%	8,7%	0,0%	0,0%								

25		3,7%	4,5%	0,0%	0,0%								
26		3,7%	4,5%	0,0%	0,0%								
27		1,6%	1,4%	0,0%	0,0%								
28		0,0%	0,0%	0,0%	0,0%								

Supplementary Table 2.
Primary data of RNA interference screens.

This table includes the primary data of the RNA interference screens, performed to knock-down several groups of genes of interest throughout the study: predicted-secreted and transmembrane proteins expressed from epithelia, proteins of cell-cell and cell-ECM junctions, proteins of the locomotory apparatus, proteins of the *C. elegans* HSP70 complex and allostery cues from epithelia (as defined by [1]). RNAi against GFP in CEPsh glia allows assessing the effectiveness of RNAi in CEPsh in mutant backgrounds, sensitive to the nervous system. Gene selection followed expression predictions in epithelia or glia available at [2]. L4440 is the negative control for RNAi. Cohorts denote different experimental days. Average +stdev is calculated by n=3 experimental repeats, and total
animals screened ≥100. (Italics are used in gene names as per nomenclature in *C. elegans* research.)

RNAi clones from Vidal and Ahringer are available in the RNAi libraries by as per references [3–5]
Companies Source Bioscience or Horizon.

unc-23(e25); nsIs374 subject to RNAi against Genes of the HSP70 complex						
	AVERAGE ± STANDARD DEVIATION (%)					n
Phenotypic group (glial cell length)	23-31 mm	31-38 mm	39-53 mm	54-93 mm	P value	Number of animals
**cohort 1**						
empty RNAi vector	0,0±0,0	3,5±6,0	13,8±5,0	82,8±9,6		110
hsp-1 RNAi	0,0±0,0	0,0±	0,0±0,0	0,0±	<0.0001	158
dnj-13 RNAi	96,7±1,2	3,3±1,2	0,0±0,0	0,0±0,0	<0.0001	150
dnj-19 RNAi	0,0±0,0	2,3±2,1	9,6±2,3	88,1±4,2	0.4776	126
hsf-1	99,3±1,2	0,7±1,2	0±0,0	0±0,0	<0.0001	150
unc-23(e25); nsIs374 subject to RNAi against Genes of ECM proteins Epidermis-expressed						
	AVERAGE ± STANDARD DEVIATION (%)					
Phenotypic group (glial cell length)	23-31 mm	31-38 mm	39-53 mm	54-93 mm	p-value	Number of animals
cohort 2						
empty RNAi vector	0,0±0,0	3,2±2,1	0,6±1,1	96,2±4,7		156
lam-1 RNAi	6,0±2,5	11,3±4,9	8,4±6,9	74,3±7,4	<0.0001	165
lam-2 RNAi	1,8±1,7	3,8±2,0	2,4±0,8	92,6±1,8	0.2320	163
emb-9 RNAi	12,9±4,0	30,3±2,6	6,4±1,8	50,5±4,4	<0.0001	172
unc-52 RNAi	38,3±6,5	45,3±5,3	8,8±1,1	7,6±5,0	<0.0001	132
cohort 3						
empty RNAi vector	1,2±1,1	6,8±5,3	6,4±3,1	85,6±4,5		158

epi-1 RNAi	2,0±3,5	4,8±4,4	1,1±0,9	92,1±2,1	0.0546	175
agr-1 RNAi	2,4±2,1	6,3±4,1	2,5±2,2	88,8±2,3	0.3279	161
cle-1 RNAi	4,4±1,3	9,3±4,7	3,8±2,0	82,4±5,3	0.2293	159
fbl-1 RNAi	0,6±1,0	3,6±3,2	1,9±2,0	93,9±2,4	0.0752	160
clec-1 RNAi	0,0±0,0	2,4±2,6	1,1±2,0	96,5±4,5	0.0044	160
cohort 4						
empty RNAi vector	0,6±1,1	3,2±1,2	5,8±1,8	90,3±4,2		155
let-2 RNAi	1,9±2,0	0,6±1,1	5,8±1,8	91,6±4,4	0.3653	154
mig-6 RNAi	0,7±1,2	1,9±1,9	1,9±1,9	95,5±2,9	0.1943	158
pxn-2 RNAi	1,8±1,9	3,6±5,0	6,6±3,0	88,0±2,8	0.8540	169
dgn-1 RNAi	0,0±0,0	1,7±1,6	8,1±2,0	90,2±1,2	0.5208	173
cohort 5						
empty RNAi vector	0,0±0,0	8,0±4,0	33,3±11,5	58,7±12,2		75
him-4 RNAi	0,0±0,0	13,3±10,1	24,0±10,6	62,7±12,2	0.3713	75
unc-23(e25); nsIs374 subject to RNAi against Genes of Predicted Epidermis Secreted Proteins						
	AVERAGE ± STANDARD DEVIATION (%)					
Phenotypic group (glial cell length)	23-31 mm	31-38 mm	39-53 mm	54-93 mm	p-value	Number of animals
cohort 6						
empty RNAi vector	0,0±0,0	3,2±2,1	0,6±1,1	96,2±4,7		156
col-48 RNAi	13,3±2,4	16,7±5,0	6,6±3,2	62,9±6,8	<0.0001	122
adt-2 RNAi	1,7±0,1	6,4±2,8	12,1±4,9	79,8±6,6	<0.0001	175
srap-1 RNAi	5,5±2,1	3,0±2,0	7,8±3,9	84,0±2,7	<0.0001	166
col-145 RNAi	4,1±1,0	14,1±2,8	8,4±4,5	73,4±4,3	<0.0001	169
col-41 RNAi	1,8±0,1	9,1±3,4	10,2±4,7	79,0±6,5	<0.0001	167
mlt-7 RNAi	19,6±12,8	52,0±8,6	4,9±2,8	23,5±6,0	<0.0001	143
dpy-3 RNAi	9,9±6,7	35,0±8,4	10,9±5,9	44,2±11,3	<0.0001	187
col-109 RNAi	3,7±4,9	3,6±1,9	5,8±5,5	86,9±6,8	<0.0001	169
col-115 RNAi	0,7±1,2	1,3±2,2	5,9±3,9	92,1±5,8	<0.0001	155
col-104 RNAi	0,0±0,0	3,8±2,0	5,7±0,1	90,4±2,1	<0.0001	157
hog-1 RNAi	0,0±0,0	1,8±0,1	8,0±3,2	90,1±3,7	<0.0001	164
zmp-2 RNAi	0,0±0,0	2,4±1,2	5,9±1,9	91,7±2,2	<0.0001	169
dip-2 RNAi	0,7±1,1	10,0±2,5	6,7±3,5	82,6±0,6	<0.0001	161
cpz-1 RNAi	0,0±0,0	3,6±1,8	5,9±3,7	90,5±7,1	<0.0001	168
toh-1 RNAi	0,0±0,0	10,4±4,9	4,0±0,8	85,6±5,6	<0.0001	173
col-3 RNAi	0,0±0,0	0,6±1,0	9,6±3,5	89,8±4,9	<0.0001	138
aagr-4 RNAi	0,0±0,0	1,5±1,3	7,8±4,3	90,8±5,2	<0.0001	149
nas-37 RNAi	0,6±1,0	9,4±3,9	3,5±1,9	86,5±3,5	<0.0001	172
gpcp-1 RNAi	0,0±0,0	4,5±4,4	5,1±1,0	90,0±4,0	0.0411	156
dpy-5 RNAi	1,3±1,1	4,4±2,0	3,8±3,1	90,5±4,6	0.0919	155
suro-1 RNAi	0,6±1,0	3,7±1,8	5,1±3,1	90,5±2,0	0.0508	159

dgk-2 RNAi	0,6±1,0	0,6±1,0	5,3±4,9	93,5±5,6	0.0089	172
dpy-10 RNAi	0,0±0,0	4,8±3,6	4,8±2,0	90,5±5,2	0.0579	167
hpo-27 RNAi	0,0±0,0	3,7±0,4	4,9±0,9	91,4±1,0	0.065	164
C15F1.1 RNAi	0,0±0,0	4,4±2,7	5,8±3,3	89,8±3,6	0.021	155
swip-10 RNAi	0,0±0,0	3,0±2,6	5,3±4,3	91,7±5,9	0.0462	161
C52G5.2 RNAi	0,0±0,0	2,6±2,9	3,2±1,1	94,2±3,8	0.3032	154
lon-1 RNAi	0,0±0,0	1,9±0,1	3,3±3,0	94,8±3,1	0.2157	157
sym-1 RNAi	0,0±0,0	1,9±1,9	2,6±2,3	95,4±4,0	0.3162	149
sod-4 RNAi	0,0±0,0	1,4±1,2	1,3±1,1	97,3±1,1	0.5185	149
col-34 RNAi	0,7±1,2	3,2±1,1	2,6±2,9	93,6±3,1	0.5624	157
tpst-1 RNAi	0,0±0,0	0,7±1,2	0,6±1,1	98,7±1,2	0.2149	149
ace-2 RNAi	0,0±0,0	0,7±1,1	0,0±0,0	99,3±1,1	0.2147	150
clr-1 RNAi	0,5±0,9	2,4±1,1	3,5±1,5	93,6±1,3	0.2152	170
col-91 RNAi	0,0±0,0	0,7±1,1	2,4±2,7	96,9±2,8	0.1308	157
H10E21.4 RNAi	0,6±1,1	1,9±3,3	3,9±3,4	93,6±3,9	0.1383	153
col-14 RNAi	0,0±0,0	4,7±1,0	1,3±1,1	94,0±1,8	0.6012	149
faah-4 RNAi	0,0±0,0	0,7±1,2	3,3±3,0	96,0±3,4	0.0834	146
F46F11.7 RNAi	0,6±1,1	0,6±1,1	1,4±1,2	97,4±1,1	0.2924	150
col-54 RNAi	0,6±1,1	2,6±1,2	2,6±3,0	94,2±4,0	0.4406	156
fkb-5 RNAi	1,2±1,1	1,8±1,9	2,0±1,9	95,0±4,0	0.4399	160
col-65 RNAi	0,7±1,1	1,3±2,3	0,7±1,2	97,4±3,0	0.6756	137
Y47G6A.19 RNAi	0,0±0,0	0,0±0,0	1,6±2,7	98,4±2,7	0.0473	145
pamn-1 RNAi	0,7±1,2	2,0±2,0	1,3±2,3	96,0±0,0	0.6625	151
col-39 RNAi	0,0±0,0	2,9±1,9	2,9±1,1	94,2±0,8	0.3462	171
nas-7 RNAi	0,6±1,1	3,8±0,0	1,9±1,9	93,6±2,2	0.532	156
F01G10.10 RNAi	0,0±0,0	0,0±0,0	2,0±3,4	98,0±3,4	0.0245	177
snx-13 RNAi	0,0±0,0	0,7±1,1	0,0±0,0	99,3±1,1	0.2144	152
dpy-9 RNAi	0,0±0,0	2,9±2,6	1,2±1,0	95,9±2,7	>0.999	175
erp-44.2 RNAi	0,7±1,1	2,5±2,2	2,3±2,0	94,5±2,1	0.4814	167
ZC513.2	0,0±0,0	2,9±1,8	2,9±2,6	94,2±4,1	0.3461	169
Y105E8B.9 RNAi	0,6±1,1	5,2±2,9	1,8±0,2	92,3±3,9	0.3969	166
lon-3 RNAi	0,0±0,0	1,3±1,1	3,4±1,3	95,4±1,0	0.0891	174
spv-1 RNAi	0,0±0,0	2,7±3,1	4,0±2,0	93,4±5,0	0.1693	151
R07E5.4 RNAi	0,0±0,0	3,2±1,2	2,5±1,1	94,2±2,1	0.5822	158
F26E4.3 RNAi	1,1±2,0	2,5±2,6	1,6±2,8	94,7±4,6	0.5448	137
col-58 RNAi	0,0±0,0	0,0±0,0	0,6±1,0	99,4±1,0	0.0437	164
cpg-7 RNAi	1,8±1,7	1,8±1,9	1,2±1,0	95,2±2,4	0.3246	163
T19D12.6 RNAi	0,0±0,0	5,0±1,0	0,6±1,0	94,4±1,5	0.786	160
nas-23 RNAi	1,8±1,9	3,0±2,8	2,2±3,8	93,0±6,1	0.2467	164
col-162 RNAi	0,0±0,0	5,6±1,8	3,4±1,6	91,0±2,3	0.5828	176
tag-196 RNAi	0,0±0,0	1,9±1,9	4,5±2,9	93,6±3,8	0.0823	155
mnr-1 RNAi	0,0±0,0	2,4±1,3	0,6±1,0	97,0±1,2	0.8709	169
adt-1 RNAi	0,0±0,0	4,4±4,3	2,6±1,2	93,0±2,9	0.3245	157
col-183 RNAi	0,0±0,0	4,6±2,1	1,8±3,0	93,6±4,3	0.6569	175
RNAi	0,0±0,0	0,6±1,1	1,9±1,9	97,5±2,2	0.2409	155
cohort 7						
empty RNAi vector	0,0±0,0	6,7±2,3	28,0±12,0	65,3±14,0		75

col-117 RNAi	0,0±0,0	5,3±2,3	32,0±4,0	62,7±4,6	0.8576	75
col-150 RNAi	0,0±0,0	0,0±0,0	26,7±18,9	73,3±18,9	0.0713	75
mlt-11 RNAi	1,3±2,3	9,3±8,3	36,0±14,4	53,3±23,1	0.3639	75
sqt-1 RNAi	0,0±0,0	0,0±0,0	28,0±4,0	72,0±4,0	0.0886	75
cohort 8						
empty RNAi vector	1,2±1,1	6,8±5,3	6,4±3,1	85,6±4,5		158
dod-21 RNAi	1,7±1,7	7,6±2,8	5,3±0,2	85,4±3,5	0.9801	171
cohort 9						
empty RNAi vector	0,6±1,1	3,2±1,2	5,8±1,8	90,3±4,2		155
col-98 RNAi	0,7±1,1	1,8±1,8	1,9±0,1	95,6±1,6	0.1944	160
col-103 RNAi	1,9±0,1	0,6±1,0	7,6±2,3	89,9±1,0	0.2786	160
col-93 RNAi	0,0±0,0	3,1±1,1	6,9±4,6	90,0±5,3	0.9333	159
col-107 RNAi	1,3±2,2	2,4±2,8	2,4±0,9	93,9±2,0	0.4745	163
fkb-3 RNAi	3,0±2,8	4,9±2,9	8,0±6,2	84,2±5,8	0.2207	166
cohort 10						
empty RNAi vector	0,0±0,0	6,7±8,3	12,0±8,0	81,3±16,2		75
dig-1 RNAi	0,0±0,0	2,7±4,6	16,0±8,0	81,3±12,2	0.4619	75
mig-17 RNAi	0,0±0,0	9,3±2,3	16,0±4,0	74,7±6,1	0.6404	75
cohort 11						
empty RNAi vector	0,0±0,0	3,5±6,0	13,8±5,0	82,8±9,6		105
glp-1 RNAi	2,0±1,9	6,9±1,6	18,0±7,1	73,2±6,9	0.2095	116
mig-17 RNAi	0,0±0,0	2,5±2,2	2,1±3,6	95,4±4,7	0.0079	83
egl-17 RNAi	4,7±8,1	10,1±8,7	17,6±5,1	67,7±11,8	0.0058	103
unc-23(e25); nsIs374 subject to RNAi against Genes of Allostery cues						
	AVERAGE ± STANDARD DEVIATION (%)					
Phenotypic group (glial cell length)	23-31 mm	31-38 mm	39-53 mm	54-93 mm	p-value	Number of animals
cohort 12						
empty RNAi vector	0,0±0,0	3,5±6,0	13,8±5,0	82,8±9,6		110
cima-1 RNAi	0,0±0,0	1,9±3,2	0,0±0,0	98,1±3,2	<0.0001	106
egl-15 RNAi	0,0±0,0	4,5±3,0	9,0±1,1	86,5±2,2	0.003	160
unc-23(e25); nsIs374 subject to RNAi against Genes of Cell Junctions in non-sensitized background (CEPsh glia insensitive to RNAi)						
	AVERAGE ± STANDARD DEVIATION (%)					

Phenotypic group (glial cell length)	23-31 mm	31-38 mm	39-53 mm	54-93 mm	p-value	Number of animals
cohort 13						
empty RNAi vector	0,9±0.8	5,7±1.8	9,8±3.0	83,6±2.1		155
ajm-1	0,6±1,07	1,8±0,10	3,7±1,92	93,8±2,25	0.717	164
dlg-1	0,0±0,0	1,7±1,6	3,3±3,1	95,0±4,1	0.4662	166
let-413	0,0±0,0	0,6±1,1	1,3±1,1	98,1±1,9	0.0218	153
let-805	3,0±1,3	0,6±1,0	0,0±0,0	96,4±2,0	0.0002	173
mua-3	0,7±0,0	3,4±0,0	10,6±0,1	85,4±0,1	0.4421	150
unc-23(e25); nsIs374 subject to RNAi against Genes of Cell Junctions in glia-sensitized background (CEPsh glia sensitive to RNAi)						
	AVERAGE ± STANDARD DEVIATION (%)					
Phenotypic group (glial cell length)	23-31 mm	31-38 mm	39-53 mm	54-93 mm	p-value	Number of animals
cohort 14						
empty RNAi vector	11,6±0,17	16,5±0,74	13,8±0,48	58,1±0,86	<0.0001	181
ajm-1 RNAi	26,0±5	22,5±2,0	22,5±2,0	29,0±6	<0.0001	106
dlg-1 RNAi	30,4±15	24,2±8,0	22,9±8	22,4±7	<0.0001	103
let-413 RNAi	26,0±5	45,3±15,0	37,1±15	12,6±2	<0.0001	120
let-805 RNAi	14,8±2,94	29,4±2,94	29,4±2,94	26,5±1,10	0.0003	101
mua-3 RNAi	22,0±2,20	24,8±2,20	24,8±2,20	28,5±5,96	<0.0001	121
unc-23(e25);nsIs374 subject to RNAi against Genes of Locomotory Apparatus						
	AVERAGE ± STANDARD DEVIATION (%)					
Phenotypic group (glial cell length)	23-31 mm	31-38 mm	39-53 mm	54-93 mm	p-value	Number of animals
cohort 15						
empty RNAi vector	0,0±0,0	3,2±2,1	0,6±1,1	96,2±4,7		156
unc-22 RNAi	4,1±2,00	13,6±9,60	4,7±1,00	77,0±11,30	<0.0001	167
unc-112 RNAi	4,6±3,8	32,2±12,5	4,6±3,7	58,0±13,6	<0.0001	175
unc-97 RNAi	0,6±1,00	9,7±4,80	9,0±0,20	80,0±4,20	<0.0001	166
cohort 16						
empty RNAi vector	5,6±3,2	11,8±2,6	5,2±0,1	77,4±1,9		153
unc-95 RNAi	9,8±8,5	9,3±3,3	0,0±0,0	81,0±11,6	0,0094	162
deb-1 RNAi	5,8±5,4	8,5±6,6	0,0±0,0	85,7±4,6	0.0134	161
unc-45 RNAi	8,6±2,6	24,7±5,3	0,6±1,1	66,1±2,6	0.0011	165
pat-4 RNAi	13,0±7,1	24,0±9,9	0,0±0,0	63,0±17,0	< 0.0001	174
unc-98	29,9±3,9	27,6±1,5	0,6±1,0	41,9±3,3	<0.0001	174

nsIs374 wild-type animals subject to RNAi against GFP in non-sensitized background / Quantification of RNAi efficiency in CEPsh glia						
	AVERAGE ± STANDARD DEVIATION (%)					
cohort 17						
Phenotypic groups	GFP ON in 4 CEPsh	GFP OFF in 1-3 CEPsh	GFP OFF in 4 CEPsh	**P value**		Number of animals
empty RNAi vector	100,0±0,0	0,0±0,0	0,0±0,0			150
GFP RNAi	100,0±0,0	0,0±0,0	0,0±0,0	1		150
nsIs374 wild-type animals subject to RNAi against GFP in non-sensitized background / Quantification of RNAi efficiency in CEPsh glia						
	AVERAGE ± STANDARD DEVIATION (%)					
cohort 18						
Phenotypic groups	GFP ON in 4 CEPsh	GFP OFF in 1-3 CEPsh	GFP OFF in 4 CEPsh	P value		Number of animals
empty RNAi vector	100,0±0,0	0,0±0,0	0,0±0,0			150
GFP RNAi	48,2±1,6	20,4±5,5	31,4±6,5	<0.0001		93
nsIs198 wild-type animals subject to RNAi against GFP in non-sensitized background / Quantification of RNAi efficiency in CEPsh glia						
	AVERAGE ± STANDARD DEVIATION (%)					
cohort 19						
Phenotypic groups	GFP ON in 4 CEPsh	GFP OFF in 1-3 CEPsh	GFP OFF in 4 CEPsh	P value		Number of animals
empty RNAi vector	100,0±0,0	0,0±0,0	0,0±0,0			150
GFP RNAi	91,8±6,0	8,2±6,0	0,0±0,0	0.0068		1
nsIs198 wild-type animals subject to RNAi against GFP in non-sensitized background / Quantification of RNAi efficiency in AMsh glia						
	AVERAGE ± STANDARD DEVIATION (%)					
cohort 20						
Phenotypic groups	GFP ON in AMsh	GFP low in AMsh	GFP OFF in AMsh	P value		Number of animals
empty RNAi vector	100,0±0,0	0,0±0,0	0,0±0,0			100
GFP RNAi	15,6±11,3	20,7±7,1	63,8±18,2	<0.0001		100
nsIs198 wild-type animals subject to RNAi against GFP in non-sensitized background						

/ Quantification of RNAi efficiency in anterior glia						
	AVERAGE ± STANDARD DEVIATION (%)					
cohort 21						
Phenotypic groups	GFP ON in anterior glia	GFP low in anterior glia	GFP OFF in anterior glia	P value		Number of animals
empty RNAi vector	100,0±0,0	0,0±0,0	0,0±0,0			100
GFP RNAi	23,6±18,5	56,2±17,3	20,2±7,15	<0.0001		100

Supplementary Table 3. Transcripts of junctional proteins in CEPsh glial cells of L2, L4 larvae.

This table presents the expression (as a list of transcripts per million) in the CEPsh, of genes composing adherens junctions and hemidesmosomes in the CEPsh glia. Transcriptomic data arise from single-cell-RNA. References to source studies are provided.

Gene	Gene transcripts in CEPsh glia			Gene transcripts in epithelia	
	in L2 larvae	in L2 larvae	in L4 larvae	hyp4-7 of mid L2 larvae	hyp4-7 of mid L2 larvae
	transcripts per million (tpm)	adjusted tpm estimate	transcripts per million (tpm)	transcripts per million (tpm)	adjusted tpm estimate
DLG-1	275.4	260.2	265.105	77.8	58.1
AJM-1	753.3	903.8	278.437	370.9	389.6
LET-413	159.6	99	91.4	112.6	102.9
VAB-9	133.4	34.6	28.219	26.1	19.9
LET-805	56.8	26	7.791	1492	1622.4
MUA-3	109.3	85	11.122	5080.5	5661.8
MUP-4	551.6	572	102.983	2071.4	2165.3
INA-1	110.9	85.9	53.766	25	13.5
Paper reference	[2], PMID: 28818938	[2], PMID: 28818938	[6], PMID: 34237253	[2], PMID: 28818938	[2], PMID: 28818938

Supplementary Table 4. Genetically-modified animals used in this study.

This table includes the list of new mutants generated, canonical alleles available in the community, as well as unstable extra-chromosomal transgenes, stably integrated transgenes, and CRISPR knock-in strains used in this study. References to previous studies are also provided.

	Alleles and transgenes used/ Genetically modified animals used	
New mutant alleles generated in this study		
Allele	Mutation / change	Strategy
unc-23(arg5)	amino acid 340: change [Glu - to - Lys]	by EMS mutagenesis
Mutant alleles used in this study		
Allele	Mutation / change	Reference
unc-52(e444)	g/a (wild type / variant); 14657426 LGII	[7]
ina-1(gm144)	g/a (wild type / variant); 9168714 LGIII	[8]
hsp-1(ra807)	c/t (wild type / variant); 17280344 LGIV	[9]
cima-1(wy84)	g/a (wild type / variant); 7653537 LGIV	[10]
eri-1(mg366)	23bp insertion; 418238 LGIV	[11]
unc-23(e25)	g/a (wild type / variant); 8938327 LGV	[12]
egl-15(n484)	g/a (wild type / variant); 11018063 LGX	[10]
nre-1(hd20) lin-15B(hd126)	lin-15B(hd126): g/a (wild type / variant); 15729954 LGX, nre-1 not mapped	[13]
Unstable extra-chromosomal transgenes used		
Alleles	Constructs provided (in transgenic lines)	Background strains with arrays
argEx136	WRM0626bA02 fosmid; pGR357; P*elt-2*::RFP	*nsIs374; unc-23(arg5)*
argEx137	WRM0626bA02 fosmid; pGR357; P*elt-2*::RFP	*nsIs374; unc-23(arg5)*
argEx138	WRM0626bA02 fosmid; pGR357; P*elt-2*::RFP	*nsIs374; unc-23(arg5)*
argEx139	WRM0626bA02 fosmid; pGR357; P*elt-2*::RFP	*nsIs374; unc-23(arg5)*
argEx207	pSR1; P*elt-2*::RFP	*nsIs374*

argEx208	pSR1; P*elt-2*::RFP	*nsIs374*
argEx209	pSR1; P*elt-2*::RFP	*nsIs374*
argEx210	pSR4; pCM4	*nsIs374; unc-23(e25)*
argEx211	pSR4; pCM4	*nsIs374; unc-23(e25)*
argEx212	pSR4; pCM4	*nsIs374; unc-23(e25)*
argEx225	pSR10; P*elt-2*::RFP	*nsIs374; unc-23(e25)*
argEx226	pSR10; P*elt-2*::RFP	*nsIs374; unc-23(e25)*
argEx230	pSR2; P*elt-2*::RFP; pSW25	*nsIs374; unc-23(e25)*
argEx247	pSR2; P*elt-2*::RFP; pSW25	*nsIs374; unc-23(e25)*
argEx251	pSR2; P*elt-2*::RFP; pSW25	*nsIs374; unc-23(e25)*
argEx292	P*dpy-7*::Bag2; pCM4	*nsIs374; unc-23(e25)*
argEx293	P*dpy-7*::Bag2; pCM4	*nsIs374; unc-23(e25)*
argEx294	P*dpy-7*::Bag2; pCM4	*nsIs374; unc-23(e25)*
argEx262	WRM064ac06 and WRM0625dF03 fosmids; P*elt-2*::RFP; pCM4	*nsIs374; unc-23(e25)*
argEx263	WRM064ac06 and WRM0625dF03 fosmids; P*elt-2*::RFP; pCM4	*nsIs374; unc-23(e25)*
argEx264	WRM064ac06 and WRM0625dF03 fosmids; P*elt-2*::RFP; pCM4	*nsIs374; unc-23(e25)*
argEx268	WRM068cG06 fosmid; P*elt-2*::RFP; pCM4	*nsIs374; unc-23(e25)*
argEx269	WRM068cG06 fosmid; P*elt-2*::RFP; pCM4	*nsIs374; unc-23(e25)*
argEx270	WRM068cG06 fosmid; P*elt-2*::RFP; pCM4	*nsIs374; unc-23(e25)*
argEx343	pGR423; pCM4	*unc-23(e25)*
argEx352	pFC6; pGR125	*mcIs46*
argEx355	P*dpy-7*::dlg-1(antisense for RNAi); P*elt-2*::RFP; pFC5	*nsIs374; unc-23(e25)*
argEx356	P*dpy-7*::dlg-1(antisense for RNAi); P*elt-2*::RFP; pFC5	*nsIs374; unc-23(e25)*
argEx357	P*dpy-7*::dlg-1(antisense for RNAi); P*elt-2*::RFP; pFC5	*nsIs374; unc-23(e25)*
argEx366	pFC6; pGR125; *Pdpy-7::AJM-1 cDNA-YFP*	*N2*

Stably integrated transgenes used		
Allele	Constructs injected	Reference of strain
nsIs374	*pGR125*	[14]
otIs133	*Pttx-3::RFP*	[15]
nsIs198	*Pmir-228::GFP*	[14]
wyIs45	*Pttx-3::GFP::rab-3*	[10]
egIs1	*Pdat-1::GFP*	[16]

nsIs180	*Phlh-17::split-Caspase; Celo::GFP*	[17]
mcIs46	*Pdlg-1::RFP*	[18]
argIs2	*Pttx-3::mCherry::rab-3 + Phlh-17::CD4::GFP(1-10) + Pttx-3::CD4::GFP(11) + Punc-122::GFP*	*this study*
argIs9	*Pdpy-7::mKate*	*this study*
argIs5	*Pmyo-3::RFP*	*this study*
argIs6	*Prab-3::mKate-PH*	*this study*
argIs26	*pSR11(Phlh-17::myrGFP-SL2-NLS-mCherry)*	*this study*
argIs14	*pFC5(Phlh-17::myrScarlet)*	*this study*
CRISPR knock-in strains used		
	unc-52(qy80[mNG+loxP (synthetic exon)::unc-52])	[19]
	emb-9 (qy24[emb-9::mNG+loxP])	[19]

Supplementary Table 5. List of DNA vectors/ sequences used for animal transgenesis in this study. This table includes the lists of plasmids and fosmid vectors used for animal transgenesis (generated in this study or in other published studies). It also includes previously published plasmids used for subcloning or present as transgenic sequences in the previously published strains, used in this study.

List of DNA sequences used for animal transgenesis

Name	DNA sequences used for animal transgenesis	Reference study
pGR357	*Phlh-17::mKate2-PH::unc-54 UTR*	this study
pSR1	*Pmir-228::unc-23 RNAi::unc-54 UTR*	this study
pSR4	*Pdpy-7::unc-23 cDNA::unc-54 UTR*	this study
pSR11	*Phlh-17::myristoylated-GFP-SL2-NLS-mCherry::unc-54 UTR*	this study
PSR10	*Pmyo-3::unc-23 cDNA::unc-54 UTR*	this study
pCM4	*Pdpy-7::myristoylated-Cherry::unc-54 UTR*	this study
pGR423	*Pdpy7::ApiGreen::unc-54 UTR=Phlh-17::SAX_7delcyt_sfGFP*	this study
pFC5	*Phlh-17::myristoylated-Scarlet::unc-54 UTR*	this study
pCM6	*Pdpy-7::mKate::unc-54 UTR*	this study
pGR406	*Prab-3::mKate-PH*	this study
pFC6	*Phlh-17::ajm-1 cDNA-CFP*	this study
Pmyo-3::RFP	*Pmyo-3::RFP::unc-54 UTR*	[20,21]
Pelt-2::RFP	*Pelt-2::RFP::unc-54 UTR*	[22]
pSW25	*Pmir-228::mCherry::unc-54 UTR*	[23,24]
pL4440	Backbone vector for bacteria-fed RNAi	Addgene
PCR fragment	*Pdpy-7::mouse Bag2*	this study
PCR fragment	*Pdpy-7::AJM-1 cDNA-YFP*	this study
PCR fragment	*Phlh-17::dlg-1::RNAi::UTR*	this study

	Original plasmid of published strains used in this study	
Name	DNA sequences used for animal transgenesis	Reference study
pGR125	*Phlh-17::myristoylated-GFP::unc-54 UTR*	[14]

	Pmir-228::GFP::unc-54 UTR	[14]
	Pttx-3::RFP	[15]
	Pttx-3::GFP::rab-3	[15]
	Pttx-3::mCherry::rab-3 + Phlh-17::CD4::GFP(1-10) + Pttx-3::CD4::GFP(11) + Punc-122::GFP	[10]
	Pdat-1::GFP	[16]
	Phlh-17::split-Caspase; Celo::GFP	[17]
	Pdlg-1::RFP	[18]
	unc-52(qy80[mNG+loxP (synthetic exon)::unc-52])	[19]
pCW94	*odr-1pro::ajm1 cDNA:CFP*	[25]
pMH510	*gcy-5pro:SAX-7delcyt-sfGFP= gcy-5pro:ApiGreen*	[25]

	Fosmid used for animal rescue	
Name	Target gene	Reference study
WRM0626bA02	*unc-23*	Horizon Discovery
WRM064ac06	*cima-1*	Horizon Discovery
WRM0625dF03	*cima-1*	Horizon Discovery
WRM068cG06	*egl-15*	Horizon Discovery

Supplementary Table 6. Expression patterns of reporters used in this study.

This table presents the list of expression patterns of the regulatory sequences used in the plasmids providing new or previously published labeling markers, used in this study.

Expression patterns of reporters used

Promoter-regulatory sequences	Cell visualized in this study using each promoter/ regulatory sequences	Reference of expression vectors/ patterns
hlh-17	Post-embryonic CEPsh glia	[26]
mir-228	All glia: ILsh/so, OLsh/so, OLQsh/so, CEPsh/so, Amsh, ADEsh, PDEsh,PHsh	[27]
dpy-7	Embryonic and post-embryonic Hypodermal cells: hyp 1-7 cells and syncytium, P cells, and seam cells	[28]
myo-3	Embryonic and post-embryonic body-wall muscle	[20,21]
elt-2	Embryonic lineage (from 2E cell stage) and post-embryonic cells of the intestine	[22]
rab-3	99% of all neurons from embryonic (comma stage onwards) to postembryonic stages	[29,30]
ttx-3	AIY neurons in postembryonic stages	[31]
dat-1	4 bilaterally symmetric pairs of dopamine neurons (2 pairs of CEP, 1 pair of ADE, 1 pair PDE neurons) in post-embryonic stages	[16]

Supplementary Table 7. Oligonucleotide sequences used for this study

Oligo sequence (5'-3')	Use
ggaacaagaaacggtgagtcg	Genotype unc-23(arg5), sense
ccagcttagccttttcaatattcg	Genotype unc-23(arg5), antisense
cgagttatgtccggaacaag	Genotype unc-23(e25), sense
gtcaatcggtcagtgattgc	Genotype unc-23(e25), antisense
ctgtttcttagtcgcctgtac	Genotype hsp-1(ra807), sense
gttcaagattccgttggcgtc	Genotype hsp-1(ra807), antisense
caaatcctcggctccatcataa	Genotype hsf-1(sy441), sense
ccaacgcagatcctcatcaa	Genotype hsf-1(sy441), antisense
gacttccagtctgactggatcc	Genotype unc-52(e444), sense
cgtcgacacaattgcaggatgc	Genotype unc-52(e444), antisense
tttcagagctgccaagacaa	Genotype ina-1(gm144),sense
cacgggataggtcgagagtc	Genotype ina-1(gm144), antisense
ctgccacgagtaccctttagttatc	Genotype cima-1(wy84), sense
ccattccccattctgttacagacc	Genotype cima-1(wy84), antisense
gaaacccgaggacgatcctttag	Genotype egl-15(n484), sense
caatgttcgtcctgctggtagag	Genotype egl-15(n484), antisense
actgatgggtaaggaatcgaagacg	Genotype eri-1(mg366),, sense
gataaaacttcggaacatatggggc	Genotype eri-1(mg366), antisense
accacacaggcaggctcaacc	Genotype lin-15B(hd126), sense
acggaaacttaccgctggcg	Genotype lin-15B(hd126),antisense
aaaagctagcatggtctccaagggagaggctgtc	Generating plasmid pFC5
aaaagaattcttacttgtaaagctcgtccattcctccgg	Generating plasmid pFC5
tataccggtcatgcagttatgaacactgatttctcc	Generating plasmid pSR1
tatagaattccacaagagacactaaccaactcac	Generating plasmid pSR1
agtcagtgatgagatgaaaaatgtgttgaggttgagctcacggccgctatttgtatagttcatccatgc	Generating plasmid pSR4
gaacattttcaggaggacccttggagggtaccggtagaaaaaatggtctccgagctcattaacgaaaac	Generating plasmid pSR4
attaggcgcgccatgggatcatgtattggaaaagctagcag	Generating plasmid pSR11
ataagcggccgcaccggtacagcagtttccctgaatta	Generating plasmid pSR11
gaacattttcaggaggacccttggagggtaccggtagaaaaaatggtctccgagctcattaacgaaaac	Generating plasmid pGR357
gaaacgcgcgagacgaaagggcccgtacggccgactagtaggaaacagttatgtttggtatattgggaatg	Generating plasmid pGR357
gactggggtgtaagtgaatgagagg	Generating PCR Phlh-17::dlg-1::RNAi::UTR
aaacagttatgtttggtatattgggaatg	Generating PCR Phlh-17::dlg-1::RNAi::UTR

REFERENCES

1. Fan, J. *et al.* A muscle-epidermis-glia signaling axis sustains synaptic specificity during allometric growth in caenorhabditis elegans. *Elife* **9**, (2020).
2. Cao, J. *et al.* Comprehensive single-cell transcriptional profiling of a multicellular organism. *Science (1979)* **357**, (2017).
3. Rual, J. F. *et al.* Toward improving Caenorhabditis elegans phenome mapping with an ORFeome-based RNAi library. *Genome Res* **14**, (2004).
4. Kamath, R. S. & Ahringer, J. Genome-wide RNAi screening in Caenorhabditis elegans. *Methods* (2003) doi:10.1016/S1046-2023(03)00050-1.
5. Kamath, R. S. *et al.* Systematic functional analysis of the Caenorhabditis elegans genome using RNAi. *Nature* **421**, (2003).
6. Taylor, S. R. *et al.* Molecular topography of an entire nervous system. *Cell* **184**, (2021).
7. Nelson, G. A., Roberts, T. M. & Ward, S. Caenorhabditis elegans spermatozoan locomotion: Amoeboid movement with almost no actin. *Journal of Cell Biology* **92**, (1982).
8. Forrester, W. C., Perens, E., Zallen, J. A. & Garriga, G. Identification of Caenorhabditis elegans genes required for neuronal differentiation and migration. *Genetics* **148**, (1998).
9. Rahmani, P., Rogalski, T. & Moerman, D. G. The C. elegans UNC-23 protein, a member of the BCL-2-associated athanogene (BAG) family of chaperone regulators, interacts with HSP-1 to regulate cell attachment and maintain hypodermal integrity . *Worm* **4**, (2015).
10. Shao, Z., Watanabe, S., Christensen, R., Jorgensen, E. M. & Colón-Ramos, D. A. Synapse Location during Growth Depends on Glia Location. *Cell* **154**, 337–350 (2013).
11. Lehner, B. *et al.* Loss of LIN-35, the Caenorhabditis elegans ortholog of the tumor suppressor p105Rb, results in enhanced RNA interference. *Genome Biol* **7**, (2006).
12. Waterston, R. H., Thomson, J. N. & Brenner, S. Mutants with altered muscle structure in Caenorhabditis elegans. *Dev Biol* **77**, (1980).
13. Schmitz, C., Kinge, P. & Hutter, H. Axon guidance genes identified in a large-scale RNAi screen using the RNAi-hypersensitive Caenorhabditis elegans strain nre-1(hd20) lin-15b(hd126). *Proc Natl Acad Sci U S A* **104**, 834–839 (2007).
14. Rapti, G., Li, C., Shan, A., Lu, Y. & Shaham, S. Glia initiate brain assembly through noncanonical Chimaerin-Furin axon guidance in C. elegans. *Nat Neurosci* **20**, (2017).
15. Wenick, A. S. & Hobert, O. Genomic cis-regulatory architecture and trans-acting regulators of a single interneuron-specific gene battery in C. elegans. *Dev Cell* **6**, (2004).
16. Nass, R., Hall, D. H., Miller, D. M. & Blakely, R. D. Neurotoxin-induced degeneration of dopamine neurons in Caenorhabditis elegans. *Proc Natl Acad Sci U S A* **99**, (2002).
17. Colón-Ramos, D. A., Margeta, M. A. & Shen, K. Glia promote local synaptogenesis through UNC-6 (netrin) signaling in C. elegans. *Science (1979)* **318**, (2007).
18. Diogon, M. *et al.* The RhoGAP RGA-2 and LET-502/ROCK achieve a balance of actomyosin-dependent forces in C. elegans epidermis to control morphogenesis. *Development* **134**, (2007).
19. Keeley, D. P. *et al.* Comprehensive Endogenous Tagging of Basement Membrane Components Reveals Dynamic Movement within the Matrix Scaffolding. *Dev Cell* **54**, (2020).
20. Rapti, G., Richmond, J. & Bessereau, J.-L. A single immunoglobulin-domain protein required for clustering acetylcholine receptors in C. elegans. *EMBO J* **30**, 706–718 (2011).

21. Gendrel, M., Rapti, G., Richmond, J. E. & Bessereau, J.-L. A secreted complement-control-related protein ensures acetylcholine receptor clustering. *Nature* **461**, 992–996 (2009).
22. Fukushige, T., Hawkins, M. G. & McGhee, J. D. The GATA-factor elt-2 is essential for formation of the Caenorhabditis elegans intestine. *Dev Biol* **198**, 286–302 (1998).
23. Wallace, S. W., Singhvi, A., Liang, Y., Lu, Y. & Shaham, S. PROS-1/Prospero Is a Major Regulator of the Glia-Specific Secretome Controlling Sensory-Neuron Shape and Function in C. elegans. *Cell Rep* **15**, 550–562 (2016).
24. Wallace, S. W. *et al.* Nuclear hormone receptors promote gut and glia detoxifying enzyme induction and protect C. elegans from the mold P. brevicompactum. *Cell Rep* **37**, (2021).
25. Low, I. I. C. *et al.* Morphogenesis of neurons and glia within an epithelium. *Development (Cambridge)* **146**, (2019).
26. Yoshimura, S., Murray, J. I., Lu, Y., Waterston, R. H. & Shaham, S. mls-2 and vab-3 Control glia development, hlh-17/Olig expression and glia-dependent neurite extension in C. elegans. *Development* **135**, 2263–2275 (2008).
27. Pierce, M. L. *et al.* MicroRNA-183 family conservation and ciliated neurosensory organ expression. *MicroRNA-183 family conservation and ciliated neurosensory organ expression* **10**, 106–113 (2008).
28. Gilleard, J. S., Barry, J. D. & Johnstone, I. L. cis Regulatory Requirements for Hypodermal Cell-Specific Expression of the Caenorhabditis elegans Cuticle Collagen Gene dpy-7 . *Mol Cell Biol* **17**, (1997).
29. Nonet, M. L. *et al.* Caenorhabditis elegans rab-3 mutant synapses exhibit impaired function and are partially depleted of vesicles. *The Journal of Neuroscience* **17**, 8061–8073 (1997).
30. Stefanakis, N., Carrera, I. & Hobert, O. Regulatory Logic of Pan-Neuronal Gene Expression in C. elegans. *Neuron* **87**, 733–750 (2015).
31. Hedgecock, E. M., Culotti, J. G., Thomson, J. N. & Perkins, L. A. Axonal guidance mutants of Caenorhabditis elegans identified by filling sensory neurons with fluorescein dyes. *Dev Biol* **111**, 158–170 (1985).
