# Supplementary material for: Age-progressive interplay of HSP-proteostasis, ECM-cell junctions and biomechanics ensures C. elegans astroglial architecture
Source: Nat Commun. 2024 Apr 3;15:2861. doi: 10.1038/s41467-024-46827-2 (PMC10991496; doi:10.1038/s41467-024-46827-2)
Supplement: Supplementary file 3 — Description of Additional Supplementary Files [file 41467_2024_46827_MOESM3_ESM.pdf]

Description of Additional Supplementary Files

File Name: Supplementary Movie 1
Description: CEPsh glia membrane sheath associates with the epidermis in *C. elegans* larvae. Physical interaction between CEPsh glia (green) and the epidermis (magenta), shown by volumetric reconstruction of confocal images of tissues in L4 larval stage. The latter part focuses on the interaction of axonassociated posterior CEPsh membranes with the epidermis. Molecular reporters used as listed in Methods and Supplementary Tables 4, 5.

File Name: Supplementary Movie 2
Description: CEPsh glia membranes appose to the body wall muscle in *C. elegans* larvae. Physical interaction between CEPsh glia (green) and the body wall muscles (magenta), shown by volumetric reconstruction of confocal images of the tissues in L4 larval stage. The latter part focuses on the interaction of axon-associated posterior CEPsh membranes with the muscle. Molecular reporters used as listed in Methods and Supplementary Tables 4, 5.

File Name: Supplementary Movie 3
Description: CEPsh glia membrane sheath envelops the mature brain circuit. Physical interaction between CEPsh glia (green) and the brainneuropil axons (magenta), shown by volumetric reconstruction of confocal images of the tissues in L4 larval stage. The latter part focuses on the interaction of posterior CEPsh membranes with the brain-neuropil axons. Molecular reporters used as listed in Methods and Supplementary Tables 4, 5.

File Name: Supplementary Movie 4
Description: Timelapse of CEPsh growth during the development of wild-type individuals. Growth of CEPsh glia (green) through L2, L3 and L4 stages in two wild-type individuals. Growth rates presented in Figure 2. Image alignment/centering is as described in Methods.

File Name: Supplementary Movie 5
Description: Timelapse of CEPsh growth throughout development of *unc-23* mutant individuals. Growth of CEPsh glia (green) through L2, L3 and L4 stages in two *unc-23* mutant individuals. Growth rates are presented in Figure 2. Image alignment/centering is as described in Methods.
