# Supplementary material for: Age-progressive interplay of HSP-proteostasis, ECM-cell junctions and biomechanics ensures C. elegans astroglial architecture
Source: Nat Commun. 2024 Apr 3;15:2861. doi: 10.1038/s41467-024-46827-2 (PMC10991496; doi:10.1038/s41467-024-46827-2)
Supplement: Supplementary file 9 — Reporting Summary [file 41467_2024_46827_MOESM9_ESM.pdf]

nature portfolio

Corresponding author(s): Georgia Rapti

Last updated by author(s): 20 February 2024

Reporting Summary

Nature Portfolio wishes to improve the reproducibility of the work that we publish. This form provides structure for consistency and transparency in reporting. For further information on Nature Portfolio policies, see our Editorial Policies and the Editorial Policy Checklist.

Statistics

For all statistical analyses, confirm that the following items are present in the figure legend, table legend, main text, or Methods section.

n/a	Confirmed	
☐	☒	The exact sample size (*n*) for each experimental group/condition, given as a discrete number and unit of measurement
☐	☒	A statement on whether measurements were taken from distinct samples or whether the same sample was measured repeatedly
☐	☒	The statistical test(s) used AND whether they are one- or two-sided<br>*Only common tests should be described solely by name; describe more complex techniques in the Methods section.*
☒	☐	A description of all covariates tested
☐	☒	A description of any assumptions or corrections, such as tests of normality and adjustment for multiple comparisons
☐	☒	A full description of the statistical parameters including central tendency (e.g. means) or other basic estimates (e.g. regression coefficient) AND variation (e.g. standard deviation) or associated estimates of uncertainty (e.g. confidence intervals)
☐	☒	For null hypothesis testing, the test statistic (e.g. *F*, *t*, *r*) with confidence intervals, effect sizes, degrees of freedom and *P* value noted<br>*Give P values as exact values whenever suitable.*
☒	☐	For Bayesian analysis, information on the choice of priors and Markov chain Monte Carlo settings
☒	☐	For hierarchical and complex designs, identification of the appropriate level for tests and full reporting of outcomes
☒	☐	Estimates of effect sizes (e.g. Cohen's *d*, Pearson's *r*), indicating how they were calculated

Our web collection on statistics for biologists contains articles on many of the points above.

Software and code

Policy information about availability of computer code

Data collection	Images were collected using commercial microscopes Olympus iXplore SPIN SR and Leica SP8 and their softwares CellSens and LAS X software, respectively.
Data analysis	Image analysis or rendering was performed using open-source software Fiji (ImageJ v2.9), a Python code provided in Supplementary Information file and an ImageJ script provided at the repository https://git.embl.de/grp-cba/glia-volume-measurement.

For manuscripts utilizing custom algorithms or software that are central to the research but not yet described in published literature, software must be made available to editors and reviewers. We strongly encourage code deposition in a community repository (e.g. GitHub). See the Nature Portfolio guidelines for submitting code & software for further information.

Data

Policy information about availability of data

All manuscripts must include a data availability statement. This statement should provide the following information, where applicable:

- Accession codes, unique identifiers, or web links for publicly available datasets
- A description of any restrictions on data availability
- For clinical datasets or third party data, please ensure that the statement adheres to our policy

Primary data and figures are provided in Source Data file and FigShare (as per Data Availability Statement). Further requests can be addressed to the lead author.

Research involving human participants, their data, or biological material

Policy information about studies with human participants or human data. See also policy information about sex, gender (identity/presentation), and sexual orientation and race, ethnicity and racism.

Reporting on sex and gender	Not applicable.
Reporting on race, ethnicity, or other socially relevant groupings	Not applicable.
Population characteristics	Not applicable.
Recruitment	Not applicable.
Ethics oversight	Not applicable.

Note that full information on the approval of the study protocol must also be provided in the manuscript.

Field-specific reporting

Please select the one below that is the best fit for your research. If you are not sure, read the appropriate sections before making your selection.

[x] Life sciences [ ] Behavioural & social sciences [ ] Ecological, evolutionary & environmental sciences

For a reference copy of the document with all sections, see nature.com/documents/nr-reporting-summary-flat.pdf

Life sciences study design

All studies must disclose on these points even when the disclosure is negative.

Sample size	Sample sizes for phenotypic analysis are based on standard practice in the C. elegans field (DOI: 10.1038/nn.4630, DOI: 10.1038/s41467-019-13795-x, DOI: 10.1038/s41467-021-24816-z). Sample sizes were found sufficient by sthe applied tatistical tests.
Data exclusions	Animals and images were included in data analysis and quantification, with no data exclusion, as described in Materials & Methods Section.
Replication	All experiments were performed at least 3 independent times, as indicated, comprising of independent biological replicates and yielding similar results. Independent transgnenic lines or independent days of scoring for mutant strains are treated as independent experiments for calculations of standard deviation.
Randomization	Randomization is not used in genetic experiments which are designed to compare sstrains with specific genotypes, detected by genotyping methods. Within samples of the same genotype, animals scored were randomly selected.
Blinding	Investigators performed blind scoring when possible. Blinding was not applicable when strains being compared are visibly different in phenotype, which is often the case in C. elegans genetic studies.

Reporting for specific materials, systems and methods

We require information from authors about some types of materials, experimental systems and methods used in many studies. Here, indicate whether each material, system or method listed is relevant to your study. If you are not sure if a list item applies to your research, read the appropriate section before selecting a response.

Materials & experimental systems

n/a	Involved in the study	
[x]	[ ]	Antibodies
[x]	[ ]	Eukaryotic cell lines
[x]	[ ]	Palaeontology and archaeology
[ ]	[x]	Animals and other organisms
[x]	[ ]	Clinical data
[x]	[ ]	Dual use research of concern
[x]	[ ]	Plants

Methods

n/a	Involved in the study	
[x]	[ ]	ChIP-seq
[x]	[ ]	Flow cytometry
[x]	[ ]	MRI-based neuroimaging

Animals and other research organisms

Policy information about studies involving animals; ARRIVE guidelines recommended for reporting animal research, and Sex and Gender in Research

Laboratory animals	The study involved C. elegans hermaphrodite animals from 0 day of life to 5 day of adulthood. N2 Bristol was the reference strains. All genetic backgrounds and specific strains used are listed in the Supplementary Data File. Animal phenotypes were assessed in hermaphrodites, as usual in C. elegans studies that do not study sex-specific phenomena.
Wild animals	No wild animals were involved in the study.
Reporting on sex	Animals used for phenotyping are hermaphrodites (not males), as accepted for genetic, non-sex-specific studies of the hermaphroditic species Caenorhabditis elegans.
Field-collected samples	No field-collected animals were involved in the study.
Ethics oversight	Followed as per EMBL and German rules and regulations (ethics approval is not required for the use of C. elegans).

Note that full information on the approval of the study protocol must also be provided in the manuscript.
